# The endogenous T cell landscape is reshaped by CAR-T cell therapy and predicts treatment response in multiple myeloma

**DOI:** 10.1038/s41375-025-02766-5

**Published:** 2025-09-19

**Authors:** Julia Frede, Julia C. Poller, Kayleen Shi, Hannah Stuart, Noori Sotudeh, Claire Havig, Klothilda Lim, Caroline R. M. Wiggers, Eugene Y. Cho, Tushara Vijaykumar, Jianlin Liu, Johannes M. Waldschmidt, Monica S. Nair, Praveen Anand, Valeriya Dimitrova, Anna Montanaro, Andrew J. Yee, Nikhil C. Munshi, Kenneth C. Anderson, Nathan Martin, Shari M. Kaiser, Marc-Steffen Raab, Noopur S. Raje, Birgit Knoechel, Jens G. Lohr

**Affiliations:** 1 Department of Medical Oncology, Jerome Lipper Multiple Myeloma Center, Dana Farber Cancer Institute, Boston, MA USA; 2 Harvard Medical School, Boston, MA USA; 3 Broad Institute of MIT and Harvard, Cambridge, MA USA; 4 Technical University of Munich, School of Medicine and Health, Institute of Clinical Chemistry and Pathobiochemistry, TUM University Hospital, Munich, Germany; 5 TUM School of Medicine and Health, Department for Internal Medicine II, Technical University of Munich, TUM University Hospital, Munich, Germany; 6 Heidelberg Myeloma Center, Department of Medicine V, University Hospital and Medical Faculty, Heidelberg University, Heidelberg, Germany; 7 Department of Pediatric Oncology, Dana-Farber Cancer Institute, Boston, MA USA; 8 Huntsman Cancer Institute, University of Utah, Salt Lake City, UT USA; 9 University of Parma, Department of Medicine and Surgery, Parma, Italy; 10 Massachusetts General Hospital, Boston, MA USA; 11 Boston VA Healthcare System, Boston, MA USA; 12 Bristol Myers Squibb, Seattle, WA USA

**Keywords:** Myeloma, Immunotherapy

## Abstract

While most patients initially respond to CAR-T cell treatment, responses often are not durable and subsequent lines of immunotherapy show diminishing success. In this study, we investigated the co-evolutionary dynamics between CAR-T cells and the immune microenvironment in myeloma patients undergoing anti-BCMA CAR-T cell therapy at single-cell resolution. Our findings highlight the transformative impact of CAR-T cell treatment on the endogenous T cell landscape. We identify a novel transitional CD8 + T cell population that is predictive of poor treatment outcomes. The emergence of this population coincides with the depletion of the endogenous T cell repertoire and compositional evolution of functional T cell subsets. These changes in the endogenous T cell compartment induced by CAR-T cell therapy may contribute to inadequate immune capacity and tumor control. Our findings highlight the potential of targeting TIM3/GAL9 interactions to mitigate T cell exhaustion, apoptosis and lack of persistence, offering promising avenues for optimizing T cell-based cancer immunotherapies. We provide a framework for assessing and manipulating the ‘mileage’ of the immune system as predictive marker and therapeutic opportunity to prevent repeated immunotherapies from becoming increasingly less successful, even when targeting distinct antigens.

## Introduction

Immunotherapies have revolutionized the treatment of blood cancers in recent years. However, durable responses are observed in only a fraction of patients. To enhance treatment outcomes, it is crucial to gain a better understanding of the molecular mechanisms underlying response and resistance to immunotherapy. Tumor-specific chimeric antigen receptor modified-T (CAR-T) cells have produced impressive responses in a subset of patients with CD19-expressing and BCMA-expressing lymphoid malignancies [1, 2]. However, the durability of these responses is often severely limited, and the development of treatment resistance is frequently observed [1–9]. Recent single-cell multiomic studies have begun to characterize mechanisms of CAR-T resistance in multiple myeloma, highlighting the influence of both CAR-T cell-intrinsic features and the immune microenvironment [10, 11]. These findings highlight the need for an improved understanding of response and resistance to CAR-T cell therapy, with the ultimate objective of improving current therapies and extending this treatment approach to other diseases.

Several cellular mechanisms reduce the efficacy of CAR-T cell treatment and promote therapeutic resistance, including inefficient trafficking into the tumor site [12], limited expansion and persistence in vivo [13, 14], loss of the target antigen [15–19], as well as CAR-T cell exhaustion [20]. T cells become exhausted after prolonged exposure to antigens, leading to impaired proliferative and cytotoxic functions [20], which are associated with poor outcomes in several types of cancer [21–23]. Additionally, an immunosuppressive microenvironment can hinder CAR-T cell expansion and persistence posing further challenges to achieving durable responses [11, 24, 25].

T cells are central to the tumor microenvironment and mediate anti-tumor immune responses across various cancers. CD8 + T cells, in particular, play a crucial role in the endogenous anti-tumor response and are key targets of T cell–redirecting immunotherapies. Given the dynamic and heterogeneous nature of immune responses, longitudinal single-cell analysis of pre- and post-treatment samples has the potential to provide powerful insights into the evolution of the anti-tumor immune response.

The long-term effect of immunotherapies on endogenous anti-tumor immune responses remains to be elucidated. Considering the increasing importance of CAR-T cells in treating a growing number of malignancies, a detailed understanding of their co-evolution with other components of the immune microenvironment is needed to enhance the efficacy of CAR-T cell therapy and overcome treatment resistance. To address these challenges, we used single-cell transcriptome profiling to define the transcriptional states in CAR-T and endogenous T cells, in combination with CITE-sequencing and VDJ sequencing. Focusing on anti-BCMA CAR-T cells in multiple myeloma patients from the pivotal KarMMa-2 and -3 trials, we investigated the interactions that influence CAR-T cell function and treatment resistance. Our study reveals that specific endogenous CD8 + T cell states correlate with therapy response, including a novel transitional population linked to poor outcomes. Our findings illustrate how CAR-T cell treatment reshapes the T cell landscape, potentially hindering effective immune responses over time. This work paves the way for identifying new immune modulatory targets and enhancing T cell–based cancer therapies.

## Methods

Detailed descriptions of all experimental procedures are provided in the Supplementary Material. In brief, peripheral blood and bone marrow samples were collected longitudinally from 24 multiple myeloma patients treated with ide-cel CAR-T cell therapy in the KarMMa-2 (NCT03601078) [25, 26] and KarMMa-3 (NCT03651128) [27, 28] clinical trials (Tables 1, 2; Supplementary Table 1). Samples were obtained at three time points: prior to treatment (screening), one month post-infusion (M2D1), and six months post-infusion (M7D1). Patients were stratified into long- and short-term responders based on median progression-free survival. Samples were processed for single-cell RNA sequencing using both SMART-seq2 and 10x Genomics platforms to profile transcriptional changes in CAR-T and endogenous T cells. T cell receptor (TCR) sequencing was performed to track clonal dynamics across time points.

**Table 1:** Clinical data by outcome.

		Total (n = 24)	Long- Term Responders (*n* = 12)	Short- Term Responders (*n* = 12)	*p*-value
Age (years, mean, SD)		61.0 (9.87)	62.7 (7.66)	59.3 (11.8)	0.409
Sex (m / f, *n*, %)		18 (75%) / 6 (25%)	9 (75%) /3 (25%)	9 (75%) /3(25%)	1.00
Ethnicity (white, black or African American, *n*, %)		21 (87.5%) / 3 (12.5%)	11 (91.7%) / 1 (8.3%)	10 (83.3%) / 2 (16.7%)	0.537
Best Overall Response	Stringent complete response	12 (50%)	9 (75%)	3 (25%)	0.109
	Complete response	2 (8.8%)	1 (8.3%)	1 (8.3%)	
	Very good partial response	4 (16.7%)	2 (16.7%)	2 (16.7%)	
	Partial response	2 (8.3%)	0 (0.0%)	2 (16.7%)	
	Minimal response	1 (4.2%)	0 (0.0%)	1 (8.3%)	
	Stable disease	3 (12.5%)	0 (0.0%)	3 (25%)	
Progression Free Survival (months, mean, SD)		18.9 (10.4)	27.6 (4.96)	10.3 (6.30)	<0.001

**Table 2:** Clinical data stratified by clinical trial.

		Total (*n* = 24)	KarMMa-3 (*n* = 17)	KarMMa-2 (*n* = 7)	*p*-value
Age (years, mean, SD)		61.0 (9.87)	61.1 (9.63)	61.0 (11.0)	0.9825
Sex (m / f, n, %)		18 (75%) / 6 (25%)	13 (76%) / 4 (24%)	5 (71%) / 2 (29%)	0.7954
Ethnicity (white, black or African American, n, %)		21 (87.5%) / 3 (12.5%)	15 (88%) / 2 (12%)	6 (86%) / 1 (14%)	0.8652
Best Overall Response	Stringent complete response	12 (50%)	8 (47.1%)	4 (57.1%)	0.2825
	Complete response	2 (8.8%)	1 (5.9%)	1 (14.3%)	
	Very good partial response	4 (16.7%)	4 (23.5%)	0 (0%)	
	Partial response	2 (8.3%)	1 (5.9%)	1 (14.3%)	
	Minimal response	1 (4.2%)	0 (0%)	1 (14.3%)	
	Stable disease	3 (12.5%)	3 (17.6%)	0 (0%)	
Progression Free Survival (months, mean, SD)		18.9 (10.4)	19.2 (11.5)	18.4 (8.3)	0.8696

Computational analyses included quality control, normalization, clustering, and annotation using Seurat and Azimuth. Trajectory inference was conducted with scVelo, and clonotype tracking was performed using scRepertoire. Ligand–receptor interactions were predicted with CellPhoneDB. Functional in vitro assays were conducted to examine the effect of Galectin-9 on CAR-T cell viability using patient-derived and donor-derived T cells. Galectin-9 plasma levels were measured by ELISA to assess associations with CAR-T persistence and response.

## Results

### CAR-T cell treatment shapes the functional T cell ecosystem

**Fig. 1:**
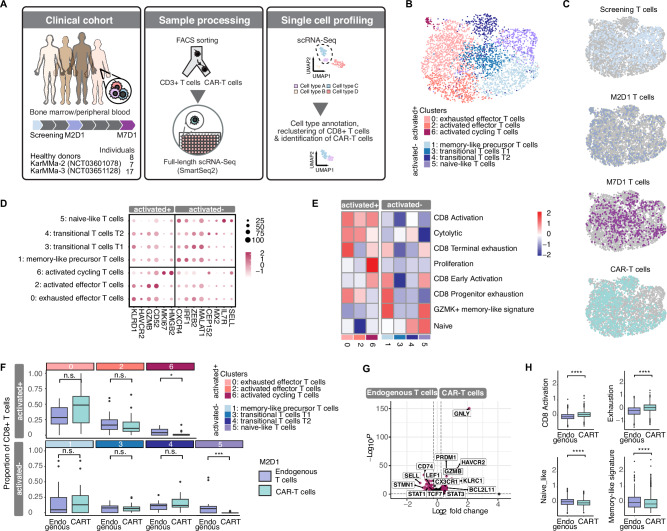
CAR-T treatment changes the endogenous T cell landscape. **A** Schematic overview of workflow. **B** UMAP of CD8 + T cells and CAR-T cells colored by clusters. **C** UMAP plots highlighting cells from each timepoint or CAR-T cells. **D** Expression of marker genes in different clusters. **E** Heatmap displaying expression of published gene signatures. **F** Comparison of proportion of cell states in endogenous T cells and CAR-T cells at M2D1 for patients. *p* = 0.11 for cluster 0, p = 0.36 for cluster 2, *p = 0.014 for cluster 6, *p* = 0.85 for cluster 1, p = 0.98 for cluster 3, *p* = 0.35 for cluster 4 and ***p = 2e-04 for cluster 5, respectively, by Wilcoxon test. **G** Volcano plot comparing endogenous CD8 + T cells at M2D1 vs. CAR-T cells. **H** Scoring of indicated signatures in endogenous CD8 + T cells at M2D1 vs. CAR-T cells. p < 2.2e − 16, p < 2.2e − 16, *p* = 4.7e − 06, *p* = 0.00031 by Wilcoxon test, respectively.

We performed single-cell full-length transcriptomics on bone marrow and blood samples from multiple myeloma patients treated with BCMA-targeted CAR-T cells (ide-cel) in the KarMMa-2 and KarMMa-3 clinical trials. Samples were collected at three time points: pre-treatment (screening), 1 month post-treatment (M2D1), and 6 months post-treatment (M7D1), from a cohort of 24 patients (Fig. 1A). To selectively enrich for rare CAR-T cells, we used fluorescence-activated cell sorting (FACS) specific for the CAR construct, followed by Smart-Seq2 [26] sequencing and quality filtering (Supplementary Fig. 1, Supplementary Table 2). To ensure high-confidence identification of CAR-T cells, we used both FACS staining and RNA expression of the CAR construct, allowing for a comprehensive comparison of CAR-T and endogenous T cells (Supplementary Fig. 2A). At the M2D1 time point, we detected 1126 CAR-T cells, while no CAR-T cells were detected at M7D1. Interestingly, CAR-T cells were almost exclusively CD8+ (Supplementary Fig. 2B). Due to the important role of CD8 + T cells in anti-tumor immunity and the low abundance of CD4 + T cells in our dataset, our analysis focused primarily on the CD8 + T cell compartment.

We retained a total of 3491 CD8 + T cells from peripheral blood (PB) and bone marrow (BM) across all timepoints (Supplementary Fig. 2C, D), including additional CD8 + T cells from 8 healthy donors for comparison. Seven distinct T cell states were defined through clustering analysis, with varying distributions across CAR-T, endogenous CD8 + T cells, and healthy donor samples (Fig. 1B–E, Supplementary Fig. 2E). Importantly, with the exception of cluster 6, which comprised actively cycling cells, clustering was not driven by sample origin or cell cycle (Supplementary Fig. 2F, G). Using published gene signatures, we categorized CD8 + T cells into activated and non-activated states (Fig. 1D, E; Supplementary Fig. 2H; Supplementary Tables 3, 4). Activated clusters included effector and cycling T cells, while non-activated clusters encompassed memory-like, transitional (T1 and T2), and naïve-like cells.

To explore differentiation states in endogenous CD8 + T cells post-treatment, we compared their functional states to those in CAR-T cells at the M2D1 time point (Fig. 1F). Both populations showed similar proportions of activated and non-activated clusters, although CAR-T cells showed fewer cycling cells and almost no naïve T cells, likely reflecting effective stimulation during manufacturing or in the recipient. Differential gene expression analysis revealed higher levels of exhaustion markers (e.g. HAVCR2/TIM3) in CAR-T cells, alongside stronger activation, exhaustion, and effector-like signatures (Fig. 1G, H), as well as expression of the bb2121 CAR construct in CAR-T cells (Supplementary Fig. 2I). In contrast, endogenous CD8 + T cells retained higher levels of memory-like and naïve-like signatures. Comparison of BM and PB samples revealed a similar distribution of cells across clusters and largely overlapping transcriptomic profiles (Supplementary Fig. 3).

These results suggest that CAR-T cells exhibit a more advanced differentiation state, characterized by increased activation and exhaustion, compared to endogenous T cells at M2D1.

### Competing differentiation states in the CD8 + T cell compartment of CAR-T-treated patients

**Fig. 2:**
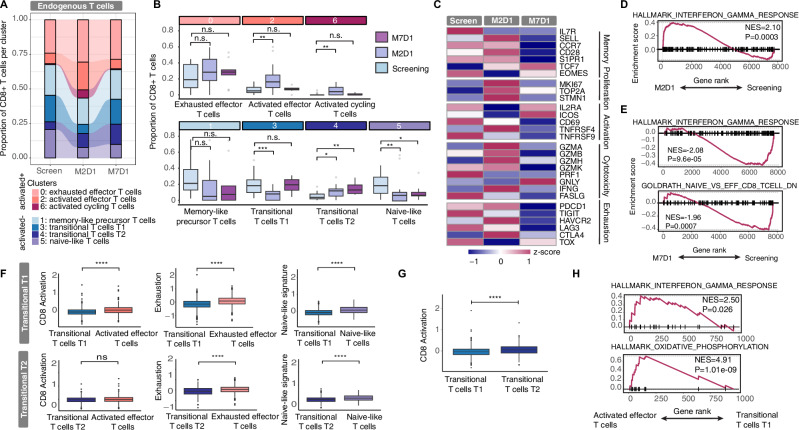
Competing differentiation states in the CD8 + T cell compartment of CAR-T-treated patients. **A, B** Proportion of clusters over time in endogenous CD8 + T cells. *p* = 0.15 and *p* = 0.29 for cluster 0, p = 0.0036 and p = 0.5 for cluster 2, *p* = 0.0065 and *p* = 0.63 for cluster 6, *p* = 0.069 and *p* = 0.15 for cluster 1, p = 0.00052 and *p* = 1 for cluster 3, *p* = 0.015 and *p* = 0.0015 for cluster 4 and *p* = 0.0025 and *p* = 0.033 for cluster 5 by Wilcoxon test, respectively. **C** Heatmap showing gene expression for representative T cell–related genes, categorized according to their function. **D** GSEA in endogenous CD8 + T cells at screening vs. M2D1. **E** GSEA in endogenous CD8 + T cells at screening vs. M7D1. **F** CD8 activation, exhaustion and naïve-like gene signature scores in transitional T cell states T1 (top) and T2 (bottom) compared to activated, exhausted and naïve CD8 + T cells. ****p = 6e − 14, ****p < 2.2e-16 and ****p < 2.2e-16 for T1 and *p* = 0.055, ****p = 7.4e-13 and ****p = 2.3e-13 for T2, respectively, by Wilcoxon test. **G** CD8 activation score. ****p = 1.2e − 08 by Wilcoxon test. **H** GSEA in transitional T cell state T1 vs. activated effector cells. For **B,F,G** box plots show the median and the interquartile range, and whiskers extend to 1.5x the interquartile range.

Having defined the differences between CAR-T cells and endogenous CD8 + T cells at M2D1, we next investigated the evolution of endogenous CD8 + T cell states over time following CAR-T cell therapy. We observed that activation in endogenous CD8 + T cells peaked at M2D1 but returned to baseline by M7D1, indicating that the activation effect induced by CAR-T therapy was transient (Fig. 2A, B). Conversely, naïve-like T cells declined at M2D1 and did not recover until M7D1, suggesting sustained depletion. The transitional subsets, T1 and T2, showed dynamic changes: T1 decreased initially and then rebounded, while T2 steadily increased over time. These shifts were supported by elevated expression of activation and proliferation markers at M2D1, followed by a decline by M7D1 (Fig. 2C–E, Supplementary Fig. 4A, Supplementary Table 5). Overall, CAR-T treatment, consisting of lymphodepletion followed by CAR-T cell infusion, appears to induce short-term activation and longer term changes in the differentiation state of endogenous T cells, marked by a reduction in naïve T cells.

We further characterized the transitional T1 and T2 states, which showed significant changes in abundance between timepoints. These cells expressed lower levels of activation, exhaustion, and naïve markers, suggesting they represent intermediate states (Fig. 2F). While T2 cells displayed higher activation compared to T1, both subsets showed low expression of cytolytic and activation markers, indicating they may not actively contribute to anti-tumor responses (Fig. 2G, H).

In summary, CAR-T cell treatment, consisting of lymphodepletion followed by CAR-T cell infusion, induces dynamic shifts in differentiation states, marked by transient activation and sustained depletion of naïve T cells in the endogenous CD8+ compartment, with the transitional T cell populations particularly showing marked changes over time.

### Association of CAR-T cell states with clinical response

**Fig. 3:**
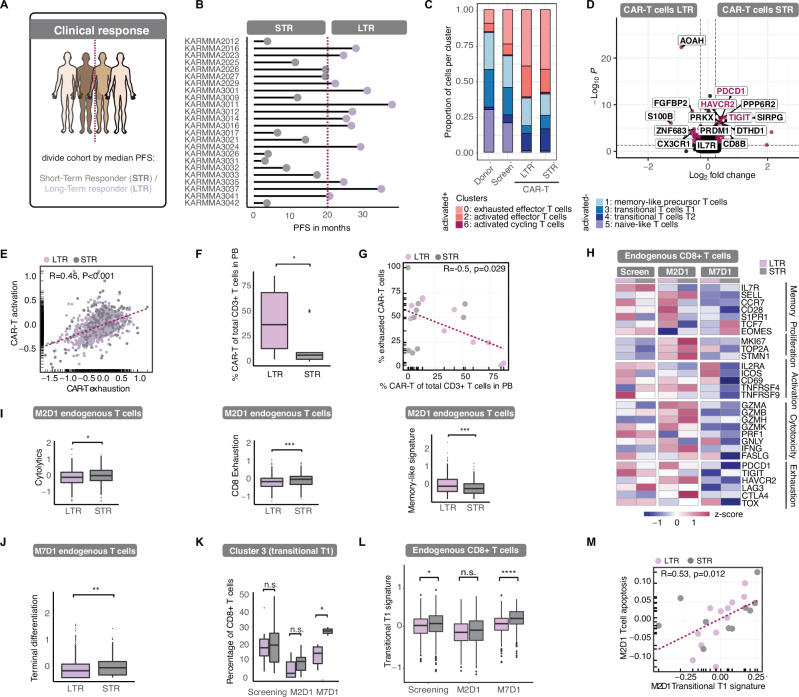
Divergent transcriptional states in T cells determine response. **A, B** Clinical response. Patient cohort was divided by median progression-free survival (PFS) into long-term responders (LTR) and short-term responders (STR) (20.14 months, red line). **C** Comparison of proportion of cells per state in normal donor CD8 + T cells, T cells from patients at screening timepoint and CAR-T cells from LTR and STR. **D** Volcano plot showing differential gene expression in CAR-T cells from LTR vs. STR. Selected canonical exhaustion markers are highlighted. **E** Pearson correlation between activation and exhaustion signatures in CAR-T cells. **F** CAR-T cell persistence in LTR vs. STR at M2D1, determined by the percentage of CAR-T cells of total CD3 + T cells in peripheral blood. Box plot shows the median and the interquartile range, and whiskers extend to 1.5x the interquartile range. p = 0.035 by Wilcoxon test. **G** Correlation of CAR-T persistence, as shown in **F** with percentage of exhausted CAR-T cells. **H** Heatmap showing gene expression in endogenous CD8 + T cells at screening, M2D1 and M7D1 in long-term responders (LTR) and short-term responders (STR) for representative T cell-related genes, categorized according to their function. **I** Gene expression scores comparing LTR and STR in endogenous CD8 + T cells at M2D1 for cytolytic markers, exhaustion markers and memory-associated markers. *p* = 0.019, *p* = 0.00064 and *p* = 0.00071 by Wilcoxon test, respectively. **J** Terminal differentiation in M7D1 endogenous CD8 + T cells. p = 0.0067 by Wilcoxon test. **K** Percentage of CD8 + T cells in transitional T cell T1 cluster (cluster 3) per timepoint by response. *p* = 0.89, *p* = 0.11 and *p* = 0.024 by Wilcoxon test for comparisons between LTR and STR, respectively. **L** Transitional T1 signature scored in endogenous CD8 + T cells in LTR and STR across timepoints. *p* = 0.0036, *p* = 0.073 and *p* = 9.1e − 09 by Wilcoxon test, respectively. **M** Correlation plot showing correlation of transitional T1 signature with apoptosis signature in endogenous CD8 + T cells.

To explore the link between different T cell states and clinical outcomes, we categorized patients into long-term (LTR) and short-term responders (STR) based on progression-free survival (PFS; Fig. 3A, B, Table 1). While the distribution of CAR-T cell states was similar between LTRs and STRs, key differences emerged compared to healthy donors and screening T cells, including a greater proportion of activated and exhausted cells and fewer naïve T cells (Fig. 3C, Supplementary Fig. 4B, C). Differential gene expression analysis revealed that STRs expressed higher levels of exhaustion (e.g., PD-1, TIM3, TIGIT) and apoptotic markers, while LTRs showed higher expression of memory-associated genes (Fig. 3D). We further found that expression of T cell activation and exhaustion signatures in CAR-T cells was positively correlated (Fig. 3E). Long-term responders exhibited higher persistence of CAR-T cells in PB at M2D1, which correlated with lower exhaustion levels (Fig. 3F, G). To contextualize our findings within a broader clinical framework, we applied the calculated PFS of 13.3 months from the KarMMa-3 study [27] to our cohort of patients (Supplementary Fig. 4D, E). This analysis confirmed consistent trends in CAR-T cell state distribution, exhaustion, terminal differentiation, and persistence (Supplementary Fig. 4F–H). Taken together, these findings suggest CAR-T cell exhaustion negatively correlates with persistence and response to CAR-T cell treatment.

### Endogenous immunity predicts anti-tumor immune response following CAR-T cell treatment

We next explored whether endogenous CD8 + T cell states could predict response to CAR-T therapy. While recent studies have linked pre-existing clonal exhausted T cells with poor response to T cell engager (TCE) therapy [28], we found no significant differences in activation or exhaustion signatures in pre-existing T cells between patients with good and poor responses to CAR-T treatment (Supplementary Fig. 4I). This suggests that the balance of activated and exhausted T cells prior to infusion does not determine treatment outcomes in our cohort of PB and BM samples from 24 patients. However, one month post-infusion (M2D1), significant differences emerged in the endogenous T cell compartment (Fig. 3H, I, Supplementary Fig. 4J). Short-term responders exhibited higher expression of both cytolytic and exhaustion markers, while long-term responders showed enhanced memory-like signatures. This indicates that persistence of endogenous T cells may correlate with better outcomes, with apoptotic factors like CASP7 also differentially regulated between groups (Supplementary Fig. 4K). At the later M7D1 timepoint, exhaustion was no longer predictive of response, though short-term responders had increased expression of terminal differentiation signatures (Fig. 3J; Supplementary Fig. 4L). Notably, effector-like and IFNγ signatures decreased in short-term responders, suggesting that initial strong activation was not maintained over time (Supplementary Fig. 4M).

We also identified differences in cell state distributions, with the transitional T cell state T1 associated with poor response at M7D1. Strikingly, short-term responders had higher expression of the T1 signature even at screening and six months post-infusion, suggesting it could serve as a predictive marker of poor clinical response (Fig. 3K,L). The T1 signature was further associated with an apoptosis signature in endogenous CD8 + T cells and was negatively correlated with CAR-T cell persistence and memory-like expression (Fig. 3M; Supplementary Fig. 4N).

In summary, our findings indicate that endogenous CD8 + T cell properties correlate with CAR-T treatment outcomes, suggesting that endogenous immunity plays a role in determining response. We identified a transitional T1 subset that is associated with poor response, suggesting its potential as a predictive biomarker.

### Sustained compositional changes in the bone marrow immune microenvironment following CAR-T cell treatment

**Fig. 4:**
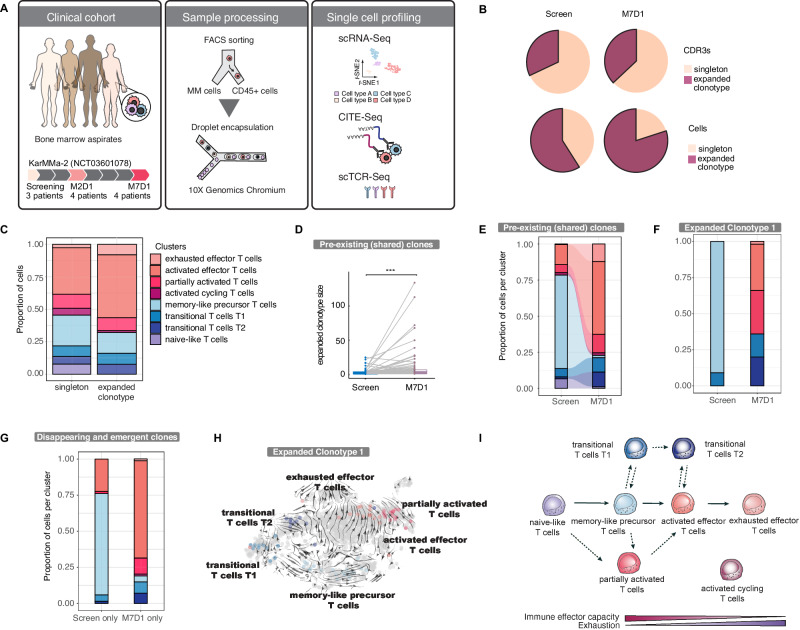
State-specific clonal expansion in the endogenous CD8 + T cell compartment of CAR-T-treated patients. **A** Schematic overview of the droplet-based single-cell RNA sequencing workflow for bone marrow aspirates. **B** Comparison of the proportion of expanded clonotypes - CDR3s (top) and cells (bottom) - at screening and M7D1 in CD8 + T cells. **C** Proportion of cell states in CD8 + T cells with singletons and expanded clonotypes. **D** Comparison of TCR clone size of clonotypes shared between screening and M7D1. ***p = 2e-05 by paired Wilcoxon test. **E** Proportions of cell states in pre-existing clonotypes, defined as clonotypes detected in screening and M7D1. **F** Proportion of cells per cell state in the individual TCR clonotype 1. **G** Stacked bar plots showing disappearing and emergent expanded clonotypes. **H** RNA velocity projected on UMAP. Cells of clonotype 1 are highlighted in color of their cell state. **I** Model of CD8 + T cell differentiation.

To characterize how CAR-T cell therapy reshapes the bone marrow immune microenvironment, we analyzed bone marrow samples from four relapsed/refractory multiple myeloma patients using droplet-based single-cell RNA sequencing and CITE-seq (Cellular Indexing of Transcriptomes and Epitopes by Sequencing) (Supplementary Fig. 5; Supplementary Table 6). Bone marrow samples were analyzed at screening, 1 month (M2D1), and 6 months (M7D1) post-infusion (Fig. 4A, Supplementary Fig. 6). We observed that CAR-T cell treatment initially skewed the bone marrow composition towards the myeloid/monocytic compartment, likely due to lymphodepletion, as seen by an increase in CD14+ monocytes at M2D1 (Supplementary Fig. 6F). Conversely, CD8+ effector cells decreased at M2D1 but had rebounded by M7D1 and showed a relative expansion (Supplementary Fig. 6F, G). These findings suggest that pre-treatment lymphodepletion followed by CAR-T cell therapy temporarily alters the bone marrow immune environment, shifting the balance toward myeloid cells [29].

Subclustering of 5152 endogenous CD8 + T cells from the droplet-based single-cell RNA-Seq dataset from the bone marrow of 4 patients revealed eight distinct functional states, consistent with our earlier findings, with an additional partially activated cluster (Supplementary Fig. 6H–J; Supplementary Tables 3,7). Analysis of surface protein expression using CITE-seq showed that exhausted effector cells had elevated levels of exhaustion markers (e.g., TIM3, LAG3) and inhibitory NK cell receptors, consistent with a loss of cytotoxic function. In contrast, activated effector cells exhibited high levels of markers indicative of late differentiation and limited proliferative potential. Transitional T2 cells showed greater differentiation compared to T1, marked by higher expression of activation-related proteins (Supplementary Fig. 6J, K).

To understand the changes in T cell subsets over time, we analyzed TCR clonality and its evolution following CAR-T cell therapy in our bone marrow-derived droplet-based single-cell RNA-Seq dataset. We identified 964 distinct clonotypes, allowing us to track clonal expansion. At screening, 32% of clonotypes were expanded (present in at least two cells), which increased to 37% at M7D1 (Fig. 4B). Similarly, the proportion of cells in expanded clonotypes rose from 59% to 80%, indicating enhanced clonal expansion over time. Examining clonotype distribution across T cell subsets, we found that naive cells were exclusively singletons, while activated effector subsets exhibited the highest degree of clonal expansion (Fig. 4C). Clonotype sharing between timepoints showed that 39% of clonotypes were present at both screening and M7D1, with many pre-existing clones expanding over time. The average size of shared clonotypes increased significantly from screening to M7D1 (mean = 3.3 to 10.2 cells, *p* < 0.001), primarily driven by expansion within the activated effector subset (Fig. 4D, E). This suggests that the increase in activated effector T cells was not solely due to recruitment of new clonotypes but also the expansion of existing ones. A comparison of clones shared between the screening and M2D1, and M2D1 and M7D1 timepoints to assess clonal persistence and subsequent outgrowth, revealed relative expansion of the activated effector subset followed by a contraction of cycling cells (Supplementary Fig. 7A, B).

Analysis of clonal evolution revealed that clones expanded across multiple subsets, including activated effector, transitional (T1 and T2), and memory-like precursor cells, indicating that these subsets share a common clonal origin and may differentiate from the same precursors or transdifferentiate (Fig. 4F). Further, cell cycle analysis showed no significant differences in proliferation rates among these subsets, suggesting that the expansion may arise through differentiation rather than differential proliferation (Supplementary Fig. 7C). We also observed distinct patterns for disappearing and emergent clonotypes. Expanded clonotypes that disappeared by M7D1 were predominantly memory-like precursor cells, while newly emergent expanded clonotypes at M7D1 were enriched in activated and partially activated T cells (Fig. 4G). This pattern suggests that memory-like precursor clones may not persist long-term, while new clonotypes are recruited, likely from the periphery—a phenomenon known as clonal replacement in other cancers [30].

To explore differentiation trajectories, we performed RNA velocity analysis. Naive and proliferating cells formed the root of the differentiation pathway, leading to either activated or exhausted effector T cells at the endpoints. CD8 + T cells transitioned from naive-like states through memory-like and transitional subsets (T1 and T2) or via partially activated cells towards activated effector and eventually exhausted effector states (Supplementary Fig. 7D, E). Within individual clonotypes, we noted a progression from memory-like precursors through transitional cells to activated effectors, with some pathways leading to exhaustion, suggesting flexible differentiation routes (Fig. 4H, I; Supplementary Fig. 7F). In summary, CAR-T cell therapy promotes expansion and differentiation of pre-existing clonotypes while also recruiting new clones from the periphery.

To gain broader insights into clonal diversity and its association with patient outcomes, we reconstructed TCR sequences from full-length sequencing data of 24 patient samples derived from both bone marrow (BM) and peripheral blood (PB). This analysis identified 1566 distinct clonotypes. To assess whether pre-infusional T cell clonotypes contributed to the CAR-T cell population, we performed longitudinal TCR tracking. Among 406 screening T cell clonotypes and 724 CAR-T cell clonotypes, only 30 were shared, suggesting that while some pre-existing T cell clones persisted, most clonotypes were lost during manufacturing or outcompeted post-infusion. As measures of clonotypic diversity, we assessed the proportion of singleton clonotypes and Shannon diversity (Supplementary Table 8, Supplementary Fig. 7G–J). Interestingly, CAR-T cells exhibited a greater proportion of singletons and higher shannon diversity compared to CD8 + T cells at screening (Supplementary Fig. 7G–I). Moreover, the diversity of endogenous CD8 + T cells declined significantly from M2D1 to M7D1. Notably, endogenous CD8 + T cells in long-term responders (LTRs) displayed significantly higher diversity than those in short-term responders (STRs) (Supplementary Fig. 7J). However, at M2D1, CAR-T cells in LTRs exhibited lower diversity compared to STRs. These results highlight the dynamic nature of T cell responses post-CAR-T cell infusion and suggest that diversity in endogenous CD8 + T cells, alongside selective clonal expansion in CAR-T cells, may influence therapeutic outcomes.

### GAL9 as mediator of reciprocal cell-cell interactions to promote an immunosuppressive T cell microenvironment

**Fig. 5:**
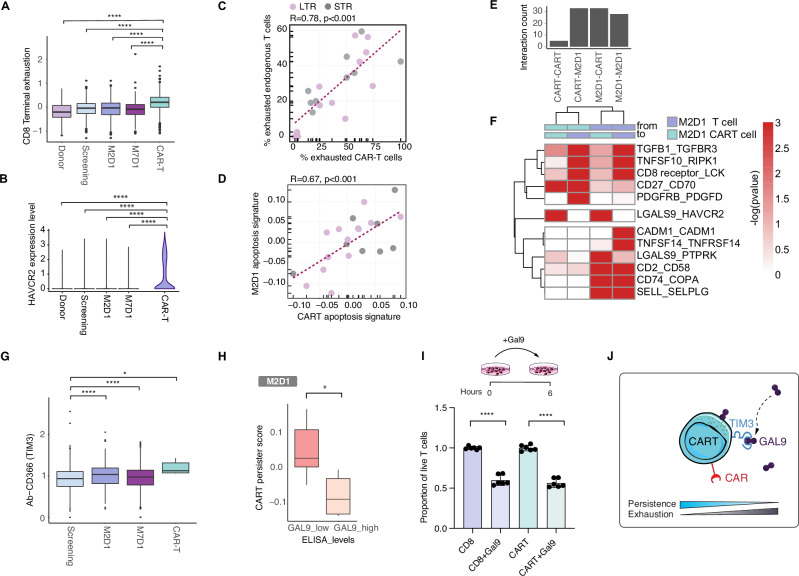
Reciprocal cell-cell interactions promote immunosuppressive microenvironment. **A** Exhaustion score. *n* = 3491 cells. ****p < 2.2e-16 by Wilcoxon test for all comparisons. **B** HAVCR2 expression. ****p < 2.2e-16 by Wilcoxon test for all comparisons. **C** Correlation between percentage of exhausted CAR-T cells and exhausted endogenous CD8 + T cells at M2D1. **D** Correlation between expression of an apoptosis gene signature in CAR-T cells and endogenous CD8 + T cells at M2D1. **E** Interaction count between CAR-T cells and endogenous T cells at M2D1. **F** Heatmap showing significant inferred cell-cell interactions between CAR-T cells and endogenous CD8 + T cells at M2D1. Only significant interactions (-log(pvalue) >3) are displayed. **G** TIM3 surface expression compared between CD8 + T cells from different timepoints and CAR-T cells generated by CITE-seq staining from the droplet-based scRNA-Seq dataset of bone marrow aspirates of 4 patients. *n* = 5158 cells. ****p = 5.9e-06 for M2D1, ****p = 3.5e-05 for M7D1 and *p = 0.026 for CAR-T cells by Wilcoxon test. **H** Boxplots showing persister scores depending on Galectin-9 plasma levels measured by ELISA. *p = 0.017 by Wilcoxon test. **I** Proportion of live T cells following pre-incubation with Gal9 in untransduced CD8 + T cells and CAR-T cells. *p* < 0.0001 by unpaired t-test. **J** Model illustrating the effect of GAL9 binding to TIM3 on CAR-T cell persistence and exhaustion. For **A,G,H** box plots show the median and the interquartile range, and whiskers extend to 1.5x the interquartile range.

Given the enrichment of CAR-T cells in the exhausted cluster, we assessed the expression of key exhaustion markers in both CAR-T and endogenous T cells with our full-length single-cell sequencing approach in PB and BM samples over time. CAR-T cells showed the highest levels of terminal exhaustion markers, including TIM3 (HAVCR2, *p* < 2.2e-16) (Fig. 5A, B), followed by endogenous T cells at M2D1. Interestingly, we found that exhaustion in CAR-T cells was correlated with exhaustion in endogenous CD8 + T cells at M2D1 (Fig. 5C). Furthermore, expression of an apoptosis signature was correlated in CAR-T cells and in endogenous CD8 + T cells (Fig. 5D). We therefore hypothesized that cell-extrinsic mechanisms and microenvironmental factors might contribute to transcriptional reprogramming and T cell exhaustion, either through direct cell-cell interactions of CAR-T and endogenous T cells, or owing to the fact that both (re-)expand in the same microenvironment following lymphodepletion.

To explore potential cell-extrinsic mechanisms contributing to T cell exhaustion, we used CellphoneDB to infer cell-cell interactions. Predicted interactions between CAR-T cells and endogenous T cells were stronger than those within each population, suggesting the potential for reciprocal signaling (Fig. 5E). We identified several receptor-ligand pairs associated with exhaustion (Fig. 5F), with a notable interaction between TIM3 on CAR-T cells and GAL9 (LGALS9, Galectin-9). This is of particular interest as GAL9 has already been suggested as an immunotherapy target [31]. To validate these interactions at the protein level, we used the CITE-seq data, confirming upregulation of TIM3 (Fig. 5G). Since GAL9 is frequently shed from the cell surface, we measured serum levels of GAL9 using ELISA and found that higher GAL9 levels were associated with lower expression of persistence-related genes in CAR-T cells, suggesting a role for GAL9 in limiting CAR-T cell persistence (Fig. 5H).

Recently, it has been proposed that the interaction of GAL9 with TIM3 may promote apoptosis [31]. To investigate this, we treated anti-BCMA CAR-T cells and untransduced normal donor CD8 + T cells with GAL9 in vitro. Both CAR-T and untransduced T cells showed a significant decrease in viability after GAL9 treatment, implying that GAL9 may contribute to reduced persistence of both CAR-T and endogenous CD8 + T cells (Fig. 5I). In a Jurkat-bb2121 CAR-T model, GAL9 treatment led to reduced viability, an effect that was partially mitigated by a GAL9-blocking antibody (Supplementary Fig. 8), supporting the idea that GAL9-TIM3 interactions promote T cell apoptosis (Fig. 5J). However, the incomplete restoration of viability (Supplementary Fig. 8B,C) suggests that additional mechanisms contribute to T cell dysfunction and apoptosis.

In summary, these findings suggest that reciprocal interactions between CAR-T cells and the bone marrow microenvironment, particularly involving GAL9, may create an immunosuppressive milieu that limits anti-tumor responses. Targeting GAL9-TIM3 interactions could therefore represent a novel therapeutic approach to enhance CAR-T cell persistence and efficacy in multiple myeloma.

## Discussion

Immunotherapies have significantly advanced cancer treatment, particularly in lymphoid diseases, with new monoclonal antibodies, bi-specific T cell engagers, and CAR-T cells being rapidly introduced. Despite their success, resistance to these therapies frequently develops, and appears to occur more rapidly with each new line of treatment [32–35], suggesting that the capacity to maintain anti-tumor immunity may diminish over time. In this study, we used single-cell sequencing technologies to investigate how CAR-T cell treatment affects the immune microenvironment. We found that CAR-T cells follow a trajectory toward terminal dysfunction and elimination, and that lymphodepletion combined with CAR-T cell infusion may contribute to a dysfunctional endogenous immune compartment. Our findings suggest that CAR-T cell therapy alters the immune landscape, which could potentially reduce its effectiveness and impact subsequent immune responses. These insights could inform the selection and sequencing of immunotherapies, such as CAR-T cells and bispecific T cell engagers (BiTEs), though further research is needed to understand these dynamics.

We defined changes in the CD8 + T cell compartment in patients treated with bb2121-BCMA CAR-T cells in the KarMMa-2 [36] and KarMMa-3 [37] trials. We observed an increase in late differentiated effector T cells and a depletion of naive-like and memory CD8 + T cells following therapy. State-specific clonal expansion was noted, suggesting that CAR-T cell therapy may promote expansion of pre-existing clones. Tracking individual clones revealed they spanned multiple cell states, implying that CD8 + T cell states, including transitional states, may share common precursor cells or transition between states. Pre-existing clones predominantly expanded within the activated effector subset, while memory-like precursor cells were more likely to disappear. Our findings highlight the dynamic nature of T cell responses post-CAR-T cell infusion, suggesting that endogenous CD8 + T cell diversity and selective clonal expansion of CAR-T cells may influence treatment outcomes. Notably, long-term responders exhibited greater endogenous CD8 + T cell diversity, whereas short-term responders showed more clonotypically diverse CAR-T cell expansion, indicating that differential clonal dynamics may be associated with treatment efficacy. However, these conclusions are based on TCR-sequencing data from a small number of patients, and thus, sampling bias cannot be ruled out. Additionally, technical constrains, such as number of cells sampled and sequencing depth, need to be taken in account when assessing the detection of recurring clonotypes.

Currently, there are no established predictive markers that reliably distinguish long-term from short-term responders to CAR-T cell therapy, despite the high costs and multiple lines of immunotherapy most patients undergo. While we identified several states of potential prognostic significance, only endogenous T1 transitional cells emerged as a population predictive of response at the time of screening. Further investigation is required to determine whether the association between these transitional cells and poor outcomes is due to their lack of effector function, or if they play an active role in negatively regulating the immune response. Preventing these detrimental changes or depleting ineffective subsets could be a strategy to enhance CD8 + T cell responses during CAR-T therapy.

Dissecting the immune interactions that influence CAR-T cell function is crucial for advancing immunotherapy [10]. In our study, we identified the GAL9-TIM3 interaction as a potential regulator of immune capacity. The GAL9-TIM3 signaling pathway is known to suppress anti-tumor immunity and contribute to T cell exhaustion [31], with GAL9 treatment of NK cells in vitro suppressing effector function [38, 39]. TIM3, which is highly expressed on CAR-T cells, is correlated with worse clinical outcomes. GAL9, produced by multiple cell types in the bone marrow [31, 40], likely plays a key role in promoting CAR-T cell exhaustion and apoptosis by binding to TIM3. Our data suggest that GAL9 could be a potential therapeutic target in multiple myeloma, as blocking this interaction might enhance CAR-T cell persistence. To validate the translational potential of targeting the Gal9/TIM3 axis, further studies inhibiting TIM3 are needed and may help determine whether T cell exhaustion in multiple myeloma is reversible. With the advent of CAR-T cell therapy and bispecific antibodies, there is renewed interest in checkpoint inhibition and as a therapeutic strategy in multiple myeloma [41–43].

Our data suggest that while CAR-T cells may eventually disappear, they leave behind an altered T cell compartment. Further research is needed to understand the long-term effects of CAR-T therapy on the bone marrow T cell niche. This may help explain why less robust responses are sometimes observed in patients undergoing subsequent rounds of CAR-T or BiTE therapy [34, 44–47]. The timing and sequencing of these immunotherapies warrant further investigation, especially as new treatments are developed.

One limitation of our study is the small number of patients and cells analyzed. While our study primarily used Smart-Seq2 for detailed full-length transcript analysis of PB and BM samples of 24 patients, we also performed 10X Genomics droplet-based immune profiling of bone marrow samples from a subset of 4 patients to complement and validate the findings. This approach allowed us to capture a broader view of the T cell landscape, addressing limitations related to cell numbers per patient. This study is focused on ide-cel and may not necessarily reflect similar associations of the T cell landscape with response to other CAR-T therapies in multiple myeloma. Additionally, all patients underwent lymphodepletion prior to CAR-T cell infusion, making it difficult to separate the effects of lymphodepletion from CAR-T cell therapy. Our findings, therefore, reflect the combined effects of the full treatment regimen, which includes both lymphodepletion and CAR-T infusion. As lymphodepletion is standard in CAR-T therapy, it remains a relevant aspect of this study. Furthermore, while we focused on T cell interactions, including the TIM3/GAL9 interaction, the potential influence of non-T cell components in the bone marrow microenvironment warrants further investigation. Furthermore, direct experimental validation of these predicted interactions, such as spatial transcriptomics or imaging-based co-localization studies, would be required to confirm their functional relevance.

In conclusion, our data suggest that CAR-T cell treatment may drive endogenous T cells along a differentiation path that initially supports a clonal anti-tumor response but could ultimately lead to terminal exhaustion and depletion. This may explain why repeated administration of CAR-T cells, even those targeting distinct antigens [45, 48], often results in diminished efficacy. Our identification of a novel transitional T cell population that may predict response at screening offers potential for further investigation into selecting patients more likely to benefit from immunotherapy. Additionally, the TIM3/GAL9 interaction emerges as a key regulator of immune capacity, with therapeutic potential for enhancing CAR-T cell persistence. These insights may in the future inform clinical decision-making and contribute to the development of more effective immunotherapies.

## Supplementary information

Supplementary Information

Supplementary Tables

## Data Availability

The sequencing data generated in this study have been deposited in the Gene Expression Omnibus (GEO) under accession number GSE230033. All other raw data needed to evaluate the conclusions in the paper are present in the paper or the Supplementary Materials. For the analysis of sequencing data, we utilized a variety of tools, including trimmomatic, STAR aligner, HTSeq, RSEM and the 10X Genomics Cell Ranger pipeline. For downstream analysis, we used R version 4.0.1 and various packages such as Seurat, mvoutlier, SingleR. All of these tools and packages are available on Github.
