# Supplementary material for: The endogenous T cell landscape is reshaped by CAR-T cell therapy and predicts treatment response in multiple myeloma
Source: Leukemia. 2025 Sep 19;39(12):3004–14. doi: 10.1038/s41375-025-02766-5 (PMC12634420; doi:10.1038/s41375-025-02766-5)
Supplement: Supplementary file 1 — Supplementary Information [file 41375_2025_2766_MOESM1_ESM.docx]

**Supplementary Information: The endogenous T cell landscape is reshaped by CAR-T cell therapy and predicts treatment response in multiple myeloma**

**Supplementary Materials**

**MATERIALS AND METHODS**

**Patient samples**

Peripheral blood and bone marrow samples from 24 patients, who had signed consent and enrolled in the KarMMa-2 (NCT03601078) [1, 2] and KarMMa-3 (NCT03651128) [3, 4] trial, were used for this study (Supplementary Table 1). These trials assess the efficacy and safety of bb2121 autologous CAR-T cell therapy in patients with relapsed and refractory multiple myeloma and high-risk multiple myeloma (KarMMa-2; NCT03601078) as well as comparing them to standard regimens (KarMMa-3; NCT03651128). Enrolled patients were at least 18 years of age and had received two to four previous therapies including daratumumab, an immunomodulatory agent, and a proteasome inhibitor and had progressed following the last therapy in KarMMa-3. KarMMa-2 was a multi-cohort study and only samples of subjects from Cohort 2 were used in our analysis. Patients of Cohort 2 have had one prior anti-myeloma treatment and showed early relapse after undergoing autologous stem cell transplantation (ASCT) in Cohort 2a, whereas patients of Cohort 2c had inadequate response to ASCT after one prior anti-myeloma treatment. Patients underwent lymphodepletion with fludarabine (30 mg per square meter of body-surface area per day) and cyclophosphamide (300 mg per square meter per day) for 3 consecutive days, followed by 2 days of rest prior to the infusion of ide-cel. Bridging therapy was allowed during the CAR-T cell manufacturing process until 14 days prior to lymphodepletion if necessary and was chosen dependent on most recent anti-myeloma treatment regimes. While in KarMMa-2 glucocorticoids, alkylating agents, immunomodulatory agents, proteasome inhibitors and/or anti-CD38 antibodies were used as single agents or in combination [1], the KarMMa-3 protocol allowed for up to 1 cycle of daratumumab/ pomalidomide/ dexamethasone (DPd), daratumumab/ bortezomib/ dexamethasone (DVd), ixazomib/ lenalidomide/ dexamethasone (IRd), carfilzomib/ dexamethasone (Kd), or elotuzumab/ pomalidomide/ dexamethasone (EPd) [3]. All the patients were followed for disease progression monthly for 24 months, then every 3 months until the occurrence of disease progression. Samples were obtained from patients before CAR-T cell treatment (at screening) as well as one month and six months after infusion (Fig. 1A). Additionally, peripheral blood samples from 8 different healthy donors were analyzed.

Ficoll-Paque density gradient centrifugation protocol was performed to extract mononuclear cells after serum separation. Cells were frozen down to a maximum of 10 million cells per 1mL and stored in liquid nitrogen until sorting. Additionally, plasma was preserved for ELISA analysis. To determine clinical significance, the patient cohort of 24 patients from KarMMa-2 and KarMMa-3 was stratified into long-term responders (LTRs) and short-term responders (STRs) by median progression-free survival. Age, sex and ethnicity were not significantly between the response groups or between KarMMa-2 and KarMMa-3 patients (p-values determined by T-test and Chi-square test; Tables 1,2). Additionally, the median PFS as calculated in Rodriguez-Otero et al. [4] was used to divide patients into LTRs and STRs in a secondary analysis.

**Single cell sorting for scRNA-seq using the SMART-seq2 protocol**

Samples were thawed, centrifuged at 1,500 rpm at 4°C and resuspended in 1% PBS-BSA before staining. Cells were stained with Fc receptor blocking solution (Human TruStain FcX, Biolegend) for 10 minutes prior to staining with antibodies against CD3 AF 647 (OKT3, Biolegend) and a specific antibody against the CAR-construct bb2121 (anti-bb2121, Bristol Myers Squibb) for 30 minutes on ice. Samples were washed 3 times with 1% PBS-BSA FACS buffer before proceeding to sort. Cells were stained with 1 μg/mL DAPI (Sigma-Aldrich) directly before sorting on a Sony Sorter SH800 machine. After gating for lymphocytes, live cells were identified based on DAPI- and doublets were excluded. Next, single cell sorting for CD3+ bb2121- cells as endogenous T cells and CD3+ bb2121+ cells as CAR-T cells was performed according to the sorting strategy (Supplementary Fig. 1A). Index sorting data was obtained for 1,282 cells from 15 patients at M2D1 for bb2121 surface/RNA-expression correlation analysis. Single cells were sorted into 96-well plates with lysis buffer cooled at 4°C and spun down immediately after sorting, followed by freezing on dry ice. Plates were then stored at -80°C until further processing.

**scRNA-seq library preparation using the SMART-seq2 protocol**

The SMART-seq2 protocol [5] was used to gain full-length single-cell RNA sequencing data from CAR-T cells and endogenous T cells in multiple myeloma patients. RNA purification, reverse transcription, PCR preamplification of cDNA and cDNA purification were performed before quality control and quantification of cDNA were performed using Qubit (Thermo Fisher Scientific) and the High Sensitivity D5000 Screen Tapes on a 2200 Tape Station System (Agilent Technologies) or the High Sensitivity DNA Kit for the 2100 Bioanalyzer System (Agilent Technologies). 0.15 ng was carried forward from each single cell and was tagmented using the Nextera XT DNA Sample Preparation Kit (Illumina). Amplification of adapter-ligated fragments, PCR purification and pooling of barcoded single cells were performed before a second quality check. Libraries were paired-end sequenced on a NextSeq 500 System (Illumina) using 75 cycle NextSeq 500 High Output v2 Kits (Illumina) aiming for an average sequencing depth of 1 million reads per cell.

**Sample preparation and scRNA-seq library preparation using the 10x Genomics platform**

Viably frozen bone marrow aspirates from four patients enrolled in the KarMMa-2 trial were thawed according to the 10x Genomics procotol ´Fresh Frozen Human Peripheral Blood Mononuclear Cells for Single Cell RNA Sequencing´. These patients were chosen for availability of longitudinal bone marrow samples. Samples of individual patients were processed by combining the timepoints of sample acquisition using oligo-tagged hashtag antibodies [6] (Biolegend). Hashing was performed before pooling samples for sorting. Cells were stained with Fc receptor blocking solution (Human TruStain FcX, Biolegend) for 10 minutes prior to staining with antibodies against CD38 FITC (multi-epitope, Cytognos; 1:200), CD138 PE (44F9, Miltenyi; 1:100), SLAMF7 APC (162.1, BioLegend; 1:100) for myeloma cells and CD45 PeCy7 (H130, Biolegend) for CD45+ immune cells (Supplementary Fig. 5A). To exclude dead cells 4,6-diamidino-2-phenylindole (DAPI; 1 μg/mL, Sigma-Aldrich) was added prior to sorting. Cell sorting was performed with a BD FACS Aria II machine. Afterwards, CD45+ immune and myeloma (on average 8% of cells) cells were pooled together for further processing. After cell sorting, the TotalSeq-C Human Universal Cocktail (BioLegend) with 200 CITE-seq [7] antibodies were added for cell surface analyses. Single-cell capture and barcoding were carried out on the 10x Genomics Chromium platform and cDNA Amplification and library preparation were performed according to 10x Genomics protocol ´Chromium Next GEM Single Cell V(D)J Reagent`. The final libraries were paired-end sequenced on an Illumina NextSeq 500 with a target reads per cell as recommended by 10x Genomics.

**ELISA**

Human Galectin-9 ELISA Kit (ab213786) by abcam was used with plasma from 11 patients at M2D1. The ELISA experiments were carried out according to the abcam protocol (version 2e) except for the first incubation step of the standards and samples on the plate. Here we incubated overnight instead of 90 minutes. O. D. absorbance was measured by microplate reader SpectraMax M5. Patients were divided by median Galectin-9 plasma concentration [pg/mL], and the CAR-T persister score was determined based on expression of a persistence-related gene signature in Gal9_low vs Gal9_high patients to assess a possible connection with CAR-T persistence.

***In vitro* CAR-T cell viability**

To assess the viability of primary CD8+ T cells and CAR-T cells following treatment with GAL9, peripheral blood from normal donors was obtained from the Crimson Core of the Brigham and Women's Hospital. Peripheral blood mononuclear cells (PBMCs) were isolated by Ficoll-Paque PLUS and CD8+ T cells were enriched using an Easysep Human CD8+ T cell isolation kit according to the manufacturer’s protocol. Isolated T cells were activated using CD3/CD28 beads (Dynabeads Human T-Activator, Thermo Fisher Scientific) and cultured in X-VIVO 15 Media (Lonza) supplemented with 5% human serum (Sigma-Aldrich) and 50 IU/mL IL2 (Miltenyi Biotec) was added every other day. One day after isolation, T cells were infected by spinoculation with lentiviral vector supernatant produced by standard methods with a CAR construct containing a single chain variable fragment targeting the BCMA antigen, derived from belantamab, cloned into a backbone comprising intracellular CD3z and costimulatory domains as detailed in *Im et al*. [8]. Uninfected T cells from the same donor were used as CD8+ T cells. To assess the effect of GAL9 on CAR-T cell viability, CAR-T and CD8+ T cells were cultured with and without recombinant Galectin-9 at 2.5 μg/mL (R&D Systems) as described previously [9]. Viability was measured using DAPI (Sigma-Aldrich) and absolute cell counts were determined based on addition of precision count beads (BioLegend) using flow cytometry 6 hours later. ﻿

**bb2121-CAR-T viability**

Jurkat cells expressing the bb2121-CAR-construct (obtained from Bristol Myers Squibb) were cultured in in RPMI-1640 (Gibco) medium with 10% fetal bovine serum, 100 μM penicillin–streptomycin (Invitrogen), 2 mM l-glutamine (Sigma), 1 mM sodium pyruvate (Sigma), 1× NEAA (Sigma), 20 mM HEPES buffer (Sigma) and 0.5 mM 2-mercaptoethanol. To assess the effect of GAL9 on CAR-T cell viability, cells were cultured with and without recombinant Galectin-9 at 2.5 μg/mL (R&D Systems) and anti-human Galectin-9 blocking antibody at 10 μg/mL (ThermoFisher Scientific) for 72 hours as described previously [9]. Viability was measured using DAPI (Sigma-Aldrich) and absolute cell counts were determined based on addition of precision count beads (BioLegend) using flow cytometry. ﻿Data were analyzed using FlowJo software v10 and Graphpad Prism v9.

**Statistical Analysis**

**Processing of plate-based single cell RNA-Seq data**

Sequencing reads were trimmed using trimmomatic and aligned to the human genome (hg19) using STAR aligner with the following specific parameters ‘-- twopassMode Basic --alignIntronMax 100000 --alignMatesGapMax 100000 --alignSJDBoverhangMin 10   --alignSJstitchMismatchNmax 5   -1   5   5’ [10, 11]. HTSeq and RSEM were utilized to acquire raw read counts and normalized TPM from the aligned bam files [12, 13].

To eliminate low-quality cells from our dataset, we employed several parameters, including the distribution of library size, the number of detected genes, the percentage of counts that map to mitochondrial genes and the percentage of counts that map to house-keeping genes per cell (Supplementary Fig. 1B,C). A cutoff of 3 median absolute deviations (MADs) was chosen, and cells that exceeded this cutoff were considered poor quality. Additionally, we used the *mvoutlier* package to identify poor quality cells without predefined cut-offs. Cells that were identified as outliers by both methods were removed from the dataset. The remaining high-quality cells were then subject to further analysis (Supplementary Table 2). We detected 353 genes in at least 50% of cells and 1281 genes in at least 25% of cells. We also investigated the impact of various technical factors, such as individual, timepoint, percentage or reads mapping to housekeeping genes and mitochondrial genes, and bb2121 detected on RNA level, on the total variation observed in the dataset and found that the contribution of these variables was low (Supplementary Fig. 1D). To ensure high-confidence identification of CAR-T cells, we used both FACS staining and RNA expression of the CAR-T construct, excluding cells that exhibited expression through only RNA (n=31) or only surface expression (n=644). Bb2121 counts were removed from the gene expression matrix and stored separately in the metadata, to avoid confounding downstream analyses focusing on endogenous transcriptional profiles.

**Clustering of single cell RNA-Seq profiles**

Seurat was used to cluster high-quality cells. Cell types were annotated using the R package *SingleR* [14] and a reference dataset of 21 immune cell populations from the BLUEPRINT [15] consortium. We selected CD8+ cells for downstream analysis and other contaminating celltypes were excluded. All available cells were pooled without downsampling to ensure maximum representation of the underlying rare cell population. We performed clustering using Seurat. A total of 7 clusters were detected with resolution = 0.5. Marker genes for each of the clusters were identified using the FindMarker function from the Seurat package.

**Calculating gene signature scores**

The CellCycleScoring function in Seurat was used to assign cell cycle phase (G1, S, and G2/M) scores to each cell. The gene signatures used in this study were derived from the Molecular Signature Database (https://www.gsea-msigdb.org/gsea/index.jsp) or defined based on the literature (Supplementary Table 3). To define cell states, we calculated scores by averaging relative expression of genesets from the literature. Heatmaps of average expression were plotted with pheatmap or the dittoHeatmap function from the dittoSeq [16] package. Fgsea [17] was used for gene-set enrichment analysis. ﻿P values were determined by one-tailed permutation test. Correlation plots were generated using smplot2 and show person correlation. A gene signature for the transitional T cells T1 was defined by determining marker genes for this cluster compared to all other clusters (Supplementary Table 4). For validation, publicly available data of CD19-CAR-T cells in B-ALL were downloaded from the ﻿European Genome-phenome Archive (accession number EGAD00001010018) and processed as described [18]. Cell type annotation was performed using azimuth and the human bone marrow dataset as a reference and CD8+ T cells were selected for comparison.

**Prediction of cellular interactions**

Cell-cell interactions between different cell types were predicted using CellphoneDB [19] (v2.0) with default settings and log-transformed normalized counts as input. The tool uses ligand-receptor co-expression to compute likelihood of interactions, which does not confirm physical co-localization or functional interaction. Interactions with p-value < 0.05 were considered significant.

**Droplet-based scRNA-seq data processing and analysis**

Raw sequencing data were processed and aligned to a custom human genome (hg19) with the bb2121 transcript using the CellRanger pipeline (10x Genomics, version 6.1.0), which efficiently distinguishes cells from empty droplets, thereby reducing the impact of ambient RNA. To further verify minimal ambient RNA contamination, we applied SoupX [20] and confirmed low contamination levels (average contamination: 2.5%; median: 1%). Red blood cell (RBC) contamination, assessed by expression of RBC-specific genes (HBA1, HBA2, HBB), was low across the dataset. The scRNA-seq data analysis of the droplet based scRNA-Seq data was conducted using the Seurat v4 package [21]. Quality control was performed on the scRNA-seq data to eliminate low-quality cells. The following criteria were used to exclude cells from the analysis: (i) Low-quality single-cell libraries with fewer than 200 detected genes or greater than 5000 detected genes or ≥ 10% mitochondrial counts or and library size < 25,000 were removed. (ii) Doublets were identified using the Scrublet Python package with the default parameters and expected_doublet_rate = 0.05, and cells with a doublet score greater than 0.2 or 0.25 were discarded.

Quality control, normalization, data integrations and clustering of single-cell transcriptomes were carried out using the Seurat v4 package [21]. The data were normalized using SCTransform. Technical or biological confounding factors (mitochondrial counts and individual) were removed by using the vars.to.regress argument in SCTransform (Supplementary Fig. 5B-D; Supplementary Table 6). Principal component analysis (PCA) was applied to reduce the data's dimensionality. The number of principal components for downstream clustering was determined using the ElbowPlot function in Seurat. The FindNeighbors function in Seurat was used to compute shared nearest neighbor graphs, and the Louvain algorithm and UMAP embedding were used to cluster cells in two-dimensional space. The ADT data were normalized using the ‘CLR’ method within Seurat and integrated with the transcriptional profiles using the *FindIntegrationAnchors* and *IntegrateData* functions. CAR-T cells were detected based on expression of the construct.

**VdJ analysis**

The cellranger vdj pipeline was used to analyze sequencing data produced from Chromium Single Cell 5′ V(D)J libraries. The pipeline was used to perform CDR3 sequence assembly and clonotype calling. 46% of CD8+ T cells had productive CDR3 chains. In this study, we define "expanded clonotypes" as T cell clonotypes that consist of more than one detected cell, as identified by their unique complementarity-determining region 3 (CDR3) sequence. Clonotypes with only one cell detected were classified as "singleton” clonotypes.

TCR sequences were reconstructed from the Smart-seq data using TRUST4 [22]. Only T cells with productive alpha (TRA) and beta (TRB) chains were included in the analysis. For each cell, the top TRAC and TRBC chains were selected based on the highest read count. scRepertoire was used for downstream analysis of clonal diversity, including clonotype definition and tracking across conditions [23].

**Cell type annotation**

Cell-type annotation was performed using azimuth (<https://app.azimuth.hubmapconsortium.org/app/azimuth-bone-marrow>) and the human bone marrow dataset as a reference. Cell type annotations were cleaned up using the *CellSelector* function in Seurat by removing cells that were annotated as one cell type but clustered with another cell type. We eliminated cell types that had a low abundance (<20 cells), which mostly consisted of progenitor populations, as well as cells annotated as ‘Early Erythroid’, ‘Late Erythroid’, ‘HSC’, ‘LMPP’ and ‘CLP’. Despite a low abundance, we retained proliferating NK cells and NKT-like CD8 effector cells. CAR-T cells were annotated based on RNA detection of the CAR-T construct.

**Differentiation trajectory**

RNA velocity/scvelo was used to identify the differentiation trajectory by analyzing the ratio of unspliced to spliced transcripts [24]. Velocity predictions were used to identify root and endpoint states and were projected onto the UMAP.

**Supplementary Figures**

Supplementary Figure 1: Quality assessment of plate-based scRNA-Seq dataset.

Supplementary Figure 2: Characterization of plate-based scRNA-Seq dataset.

Supplementary Figure 3: Comparison of peripheral blood and bone marrow at M2D1.

Supplementary Figure 4: Differential gene expression across response groups and timepoints.

Supplementary Figure 5: Quality assessment of droplet-based scRNA-Seq dataset.

Supplementary Figure 6: Characterization of droplet-based scRNA-Seq data from bone marrow cells.

Supplementary Figure 7: ﻿Clonal sharing between clusters informs differentiation.

Supplementary Figure 8: GAL9 treatment results in reduced viability in CAR-T cells.

**Supplementary Tables**

Supplementary Table 1: Patient characteristics.

Supplementary Table 2: Cell numbers for plate-based scRNA-Seq samples before and after quality filtering.

Supplementary Table 3: Gene signatures used for functional annotation of clusters.

Supplementary Table 4: Marker genes for individual CD8 T cell clusters from plate-based scRNA-Seq.

Supplementary Table 5: Marker genes for endogenous CD8+ T cells at different timepoints.

Supplementary Table 6: Cell numbers for droplet-based scRNA-Seq samples before and after quality filtering.

Supplementary Table 7: Marker genes for individual CD8 T cell clusters from droplet-based scRNA-Seq.

Supplementary Table 8: TCR clonotype singletons as determined by plate-based scRNA-Seq.

**
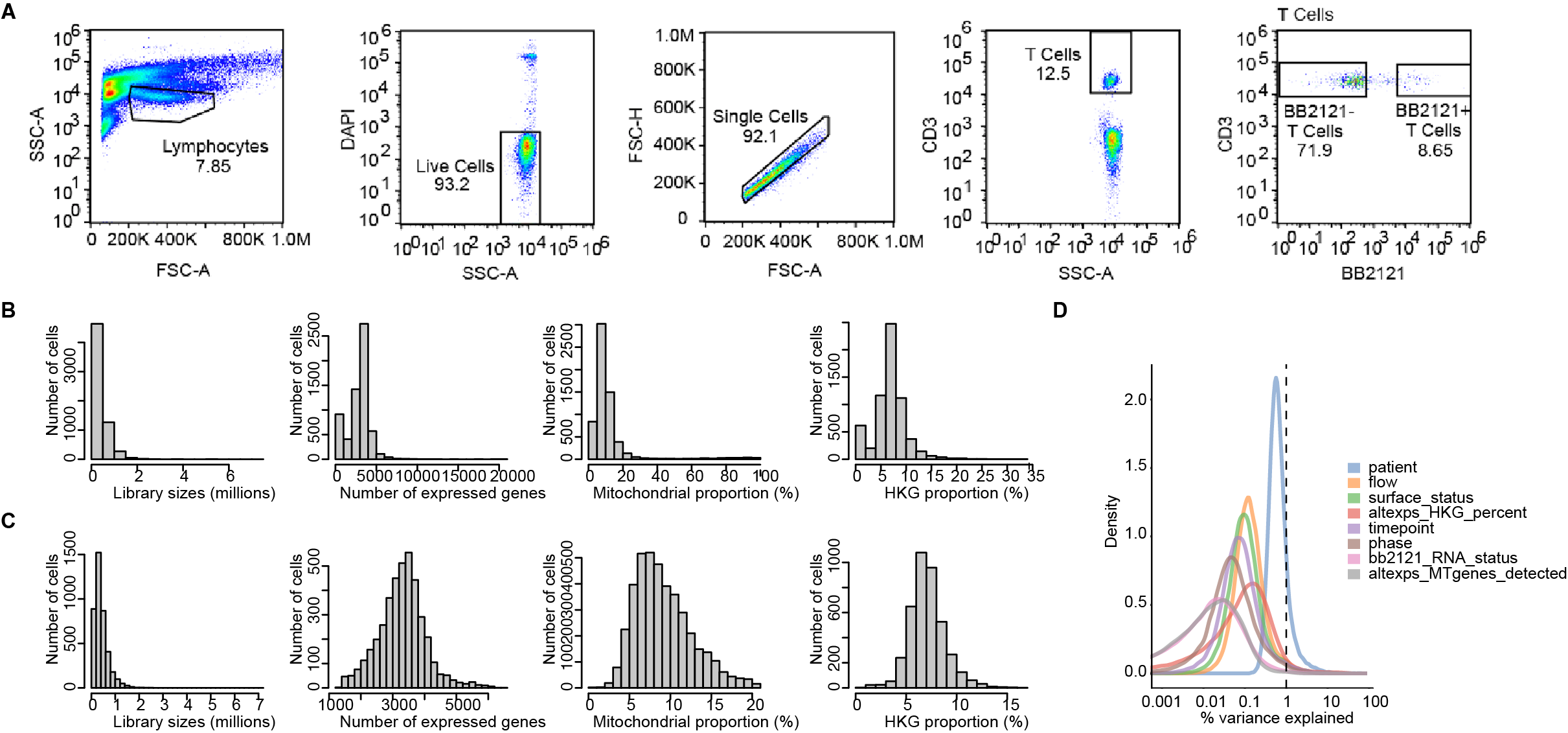
**

**Supplementary Figure 1: Quality assessment of plate-based scRNA-Seq dataset.**

**A,** Sorting strategy for CD3+bb2121- endogenous T cells and bb2121+ CAR-T cells with representative flow cytometry plots for plate-based scRNA-Seq approach. **B,** Distribution of library size, number of expressed genes, mitochondrial proportion and HKG proportion per cell before quality control filtering for plate-based scRNA-Seq approach. **C,** Distribution of features shown in **B**, after filtering. **D,** Density plot shows the contribution of various technical factors to the total variation observed in the plate-based scRNA-Seq dataset.

**
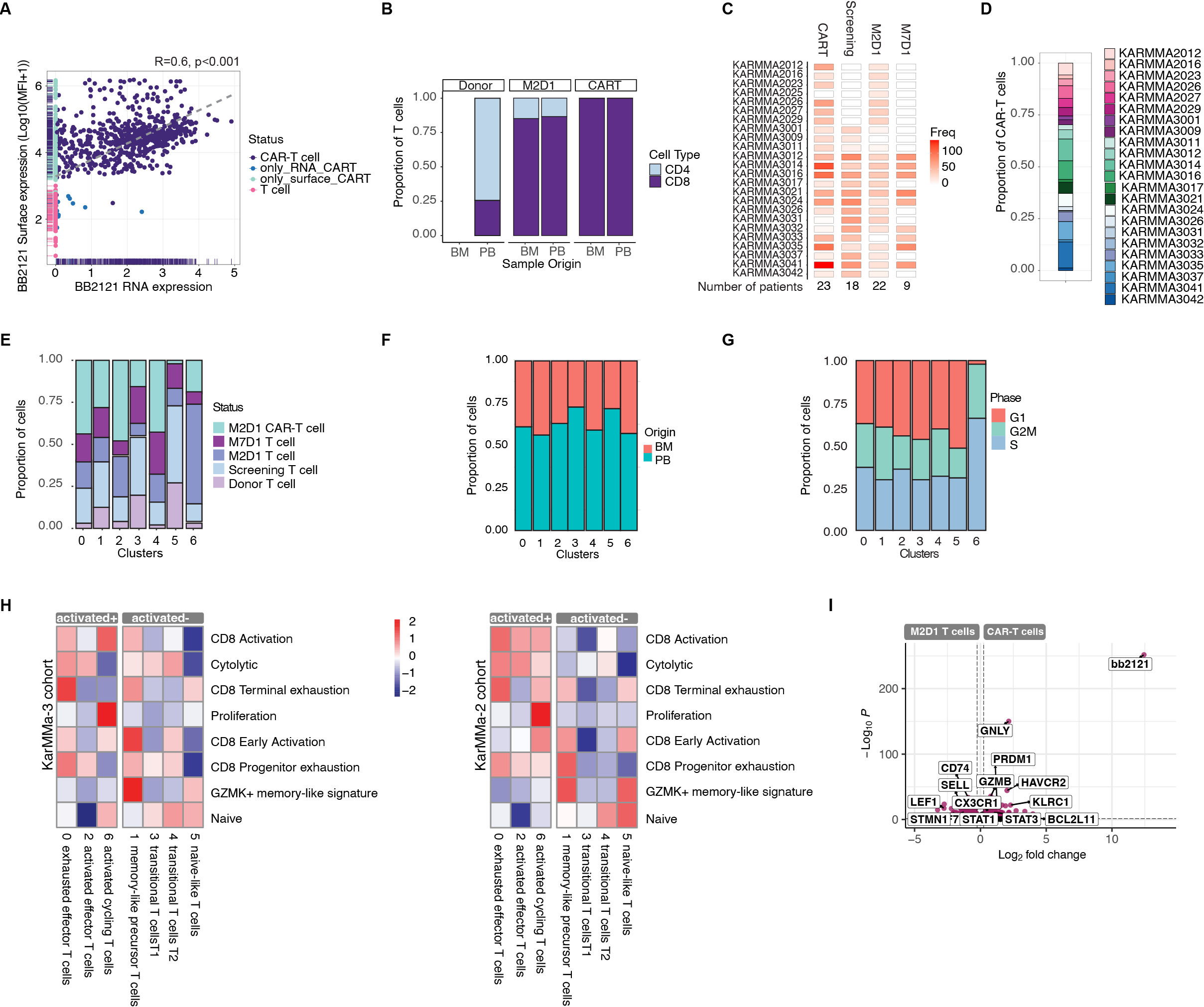
Supplementary Figure 2: Characterization of plate-based scRNA-Seq dataset.**

**A,** Correlation plot between bb2121 construct surface expression and bb2121 RNA expression. Colors show different groups: T cells, defined as negative on surface and RNA level (T cell), cells expressing bb2121 only on surface in FACS analysis (only_surface_CART), cells negative in FACS but positive on RNA level (only_RNA_CART) and cells positive in FACS and scRNAseq (CAR-T cell), which were carried forward as CAR-T cells. **B,** Proportion of CD4+/CD8+ T cells. **C,** Number of cells per individual and timepoint. **D,** Proportion of CAR-T cells at M2D1 per patient. **E,** Distribution of cells across clusters. **F,** Proportion of BM vs. PB samples in each cluster. **G,** Cell cycle phase of CD8+ T cells. **H,**Heatmaps showing expression of published gene signatures by patient cohort. **I,** Volcano plot comparing endogenous CD8+ T cells at M2D1 vs CAR-T cells including the bb2121 construct.

**
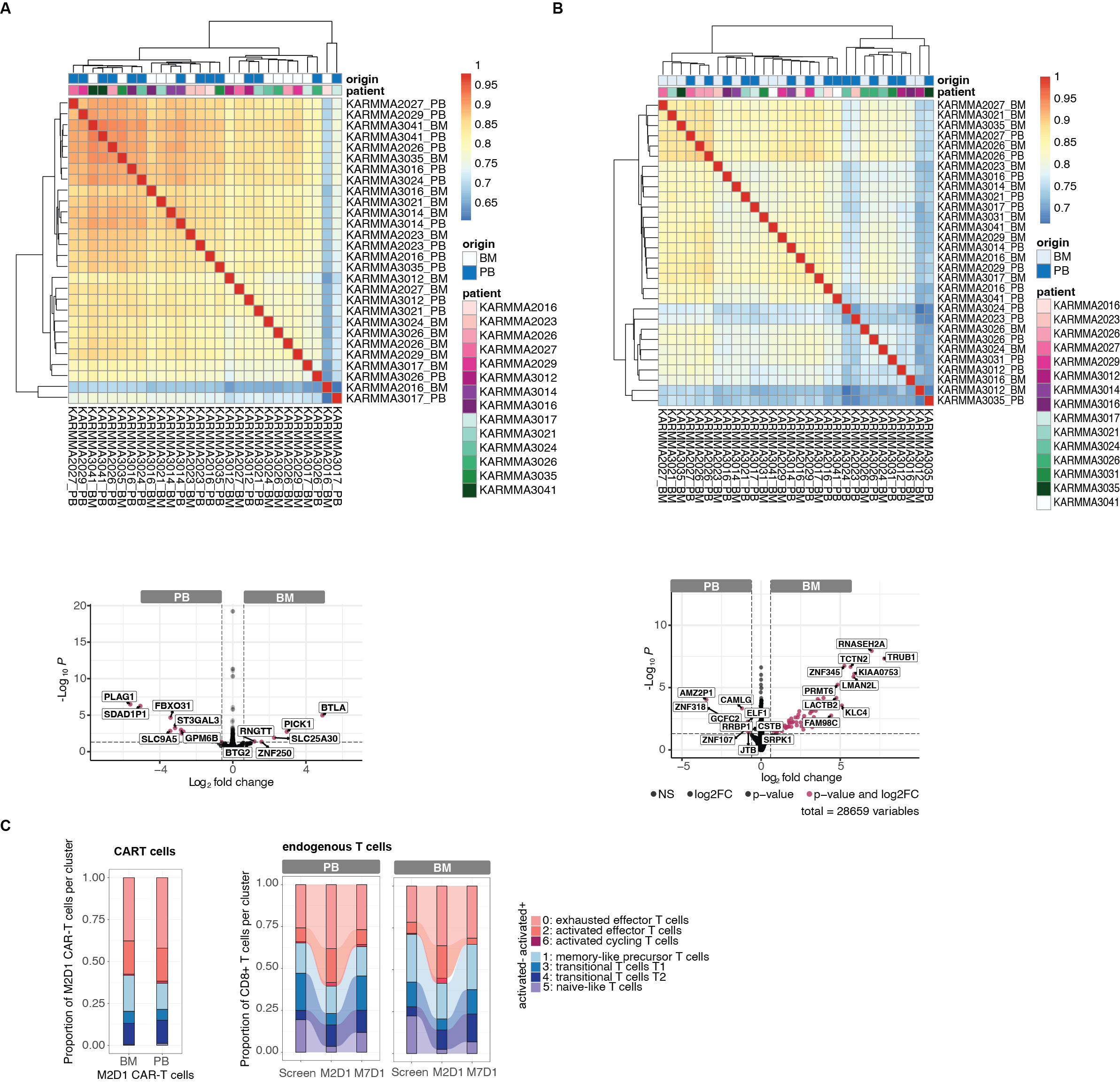
Supplementary Figure 3: Comparison of peripheral blood and bone marrow at M2D1.**

**A,** Correlation plot and volcano plot of differentially expressed genes for M2D1 CAR-T cells in patients with paired BM and PB samples. **B,** Correlation plot and volcano plot of differentially expressed genes for M2D1 endogenous CD8+ T cells in patients with pared BM and PB samples. **C,** Stacked barplots of CAR-T cells (left) endogenous CD8+ T cells (right) with proportion of cells per cluster for peripheral blood (PB) and bone marrow (BM) over time.

**
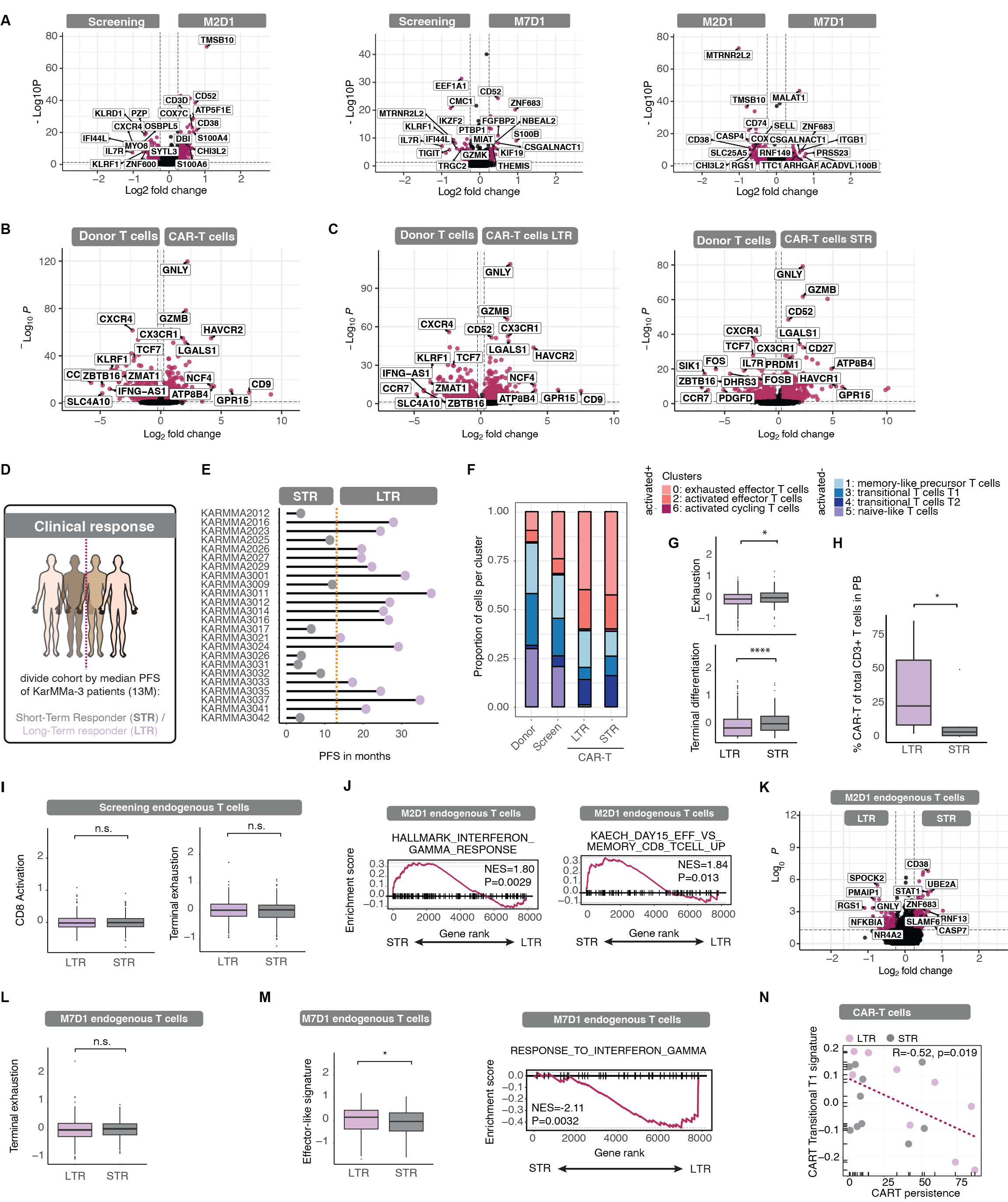
**

**Supplementary Figure 4: Differential gene expression across response groups and timepoints.**

**A,** Volcano plot showing differential gene expression between CD8+ endogenous T cells at different timepoints: Screening vs. M2D1 (left), Screening vs. M7D1 (middle) and M2D1 vs. M7D1 (right). **B,** Volcano plot showing differential gene expression between Donor T cells and all CAR-T cells at M2D1. **C,** Volcano plot showing differential gene expression between Donor T cells and LTR CAR-T cells at M2D1 (left) and between Donor T cells and STR CAR-T cells at M2D1 (right). **D,E,** Clinical response as determined in Rodriguez-Otero *et al.* for the KarMMa-3 study (median progression-free survival (PFS) of 13.3 months, orange line) applied to our patient cohort. **F**, Comparison of proportion of cells per state in normal donor CD8+ T cells, T cells at screening timepoint and CAR-T cells from LTR and STR as defined in **E**. **G,** Exhaustion score and terminal differentiation score for LTR and STR CAR-T cells as defined in **E**. n= 1124 cells. * p = 0.015, and **** p = 6e-04 by Wilcoxon test, respectively. **H,** CAR-T cell persistence in LTR vs STR at M2D1 as defined in **E**, determined by the percentage of CAR-T cells of total CD3+ T cells in peripheral blood. p = 0.026 by Wilcoxon test. **I,** Expression of activation and exhaustion signatures in endogenous CD8+ T cells at screening. **J,** Gene set enrichment analysis (GSEA) comparing endogenous CD8+ T cells at M2D1 in long-term responders (LTR) and short-term responders (STR). NES, normalised enrichment score. **K,**Volcano plot showing differentially expressed genes in endogenous CD8+ T cells at M2D1 from LTR and STR. **L,** Terminal exhaustion signature in endogenous CD8+ T cells at M7D1. p = 0.21 by Wilcoxon test. **M,**Effector-like signature in endogenous CD8+ T cells at M7D1. p = 0.031 by Wilcoxon (left) and gene set enrichment analysis (GSEA) comparing endogenous CD8+ T cells at M7D1 in LTR and STR (right). **N,** Correlation plots showing expression of the transitional T1 signature in CAR-T cells with CAR-T persistence.


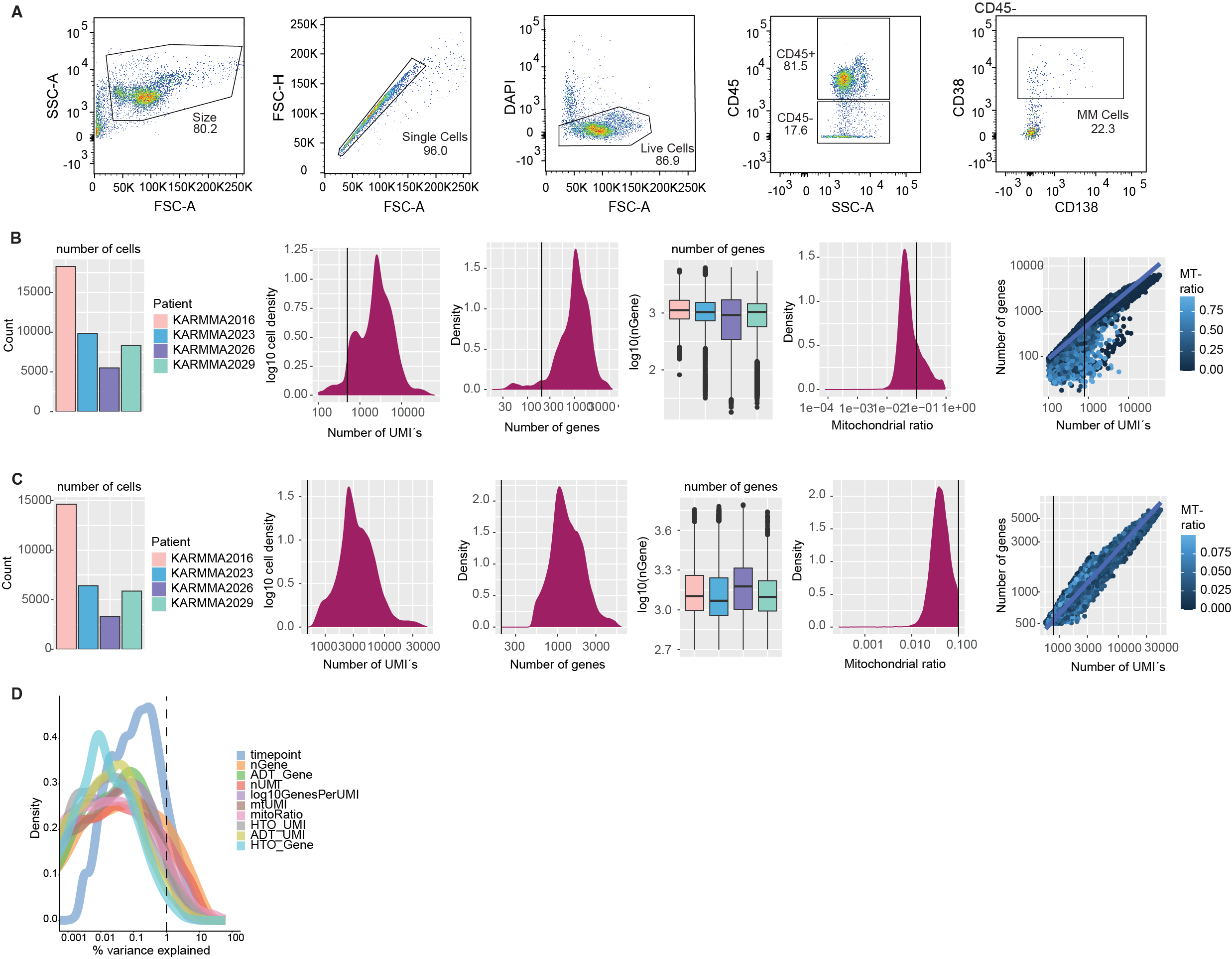


**Supplementary Figure 5: Quality assessment of droplet-based scRNA-Seq dataset.**

**A,** Sorting strategy for CD45+ immune cells and MM cells with representative flow cytometry plots for droplet-based scRNA-Seq approach. **B,** Quality control plots before filtering of droplet-based scRNA-Seq data. **C,** Distribution of features shown in (**B**) after quality control filtering. **D,** Density plot shows the contribution of various technical factors to the total variation observed in the droplet-based scRNA-Seq dataset.

**
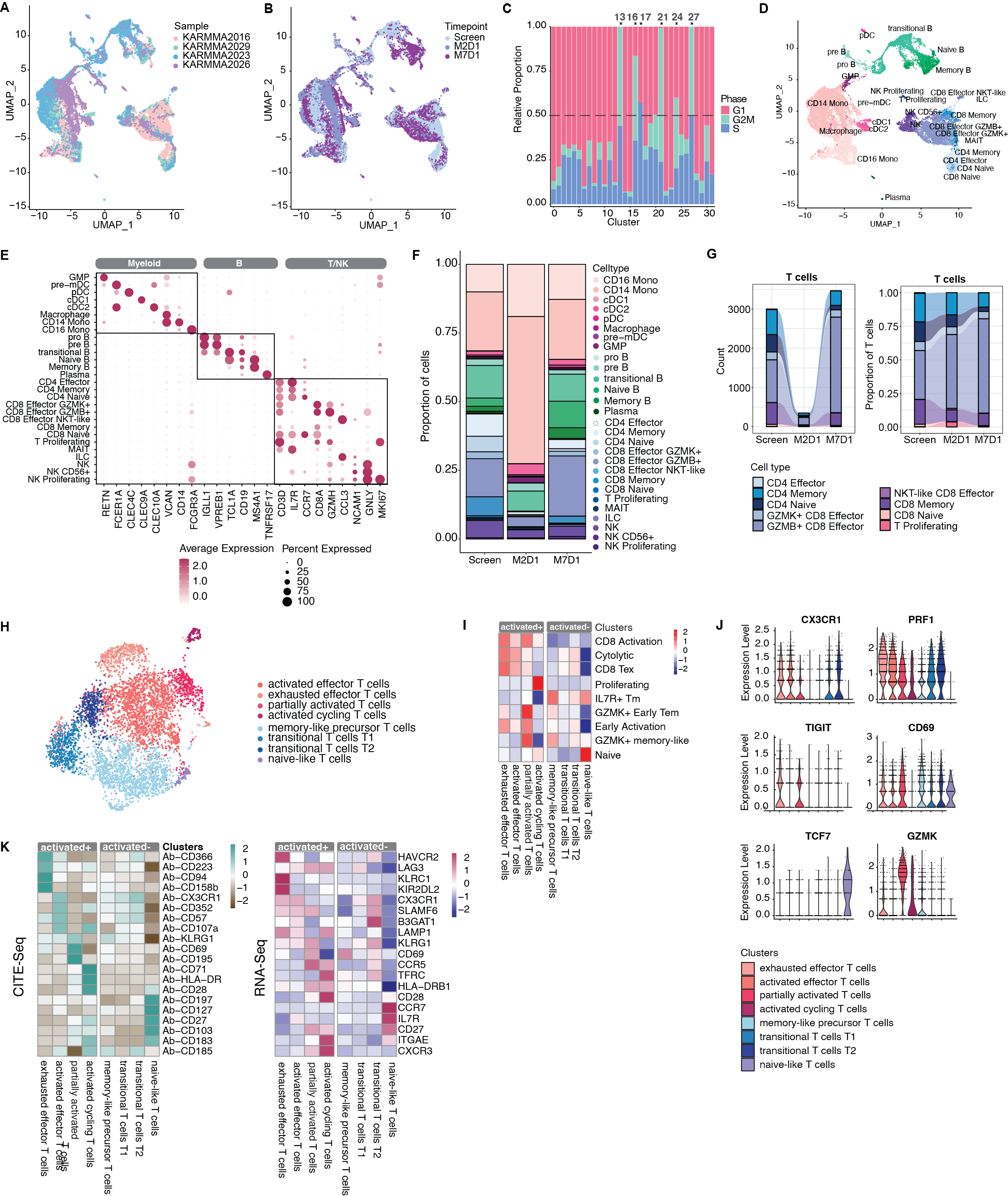
Supplementary Figure 6: Characterization of droplet-based scRNA-Seq data from bone marrow cells.**

**A,** UMAP plot color-coded by patient. **B,** UMAP plot color-coded by timepoint. **C,** Stacked bar plot showing the relative proportion of cells in each cell-cycle phase per cluster. Clusters with >50% cycling cells are marked. **D,** UMAP colored by cell type. **E,** Dotplot showing expression of marker genes for different cell types. **F,** Proportion of cell types at screening (n= 7926 cells), M2D1 (n= 5647 cells) and M7D1 (n= 11181 cells). **G,** Absolute count (left) and proportion of T cells (right). **H,** UMAP Plot of T cells. **I,** Heatmap showing expression of published gene signatures. **J,** Violin plots showing RNA expression of selected marker genes across cell states. **K,** Heatmaps showing average expression of selected surface proteins analyzed with CITEseq (left) or average RNA expression (right).

**
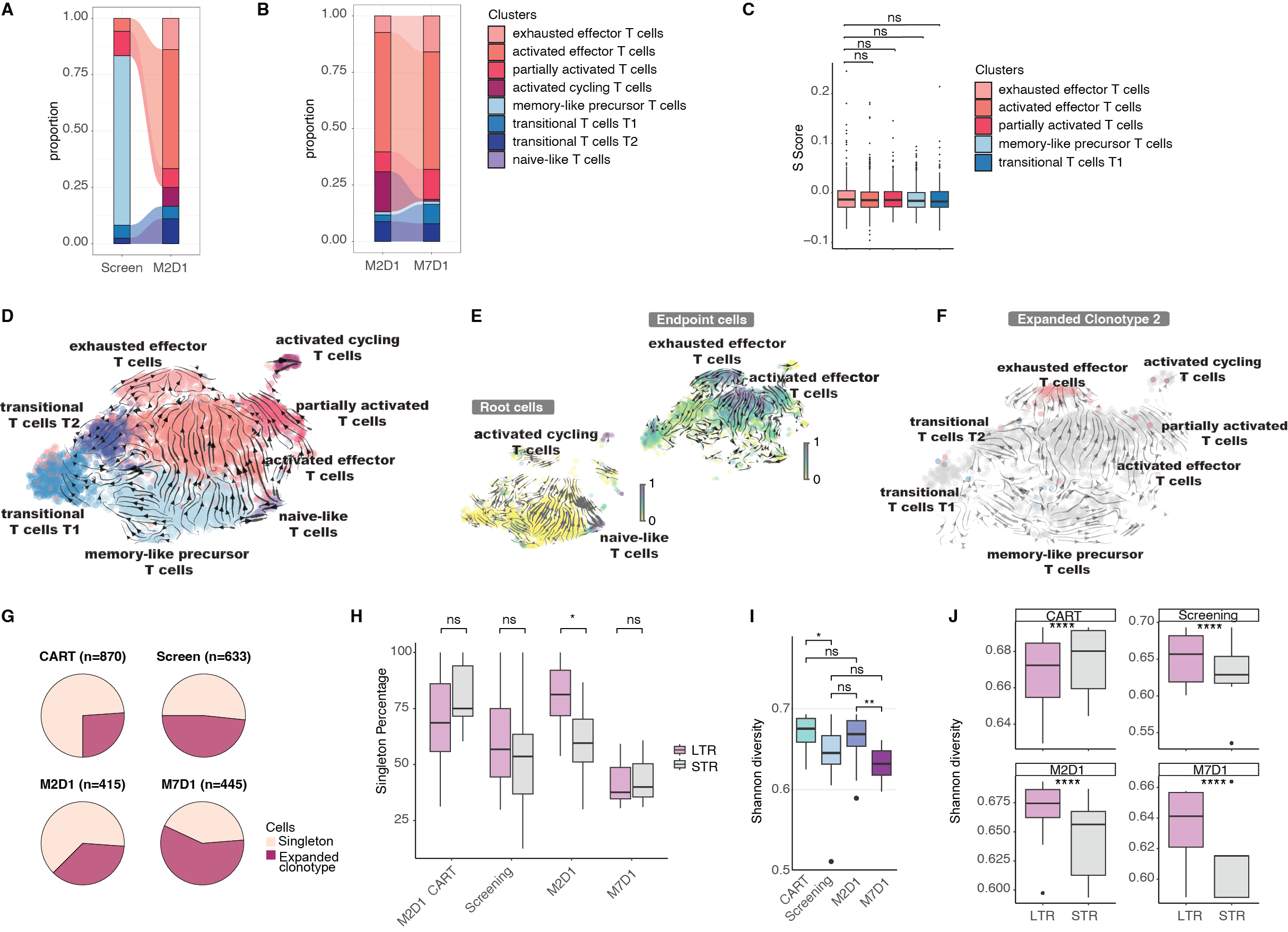
**

**Supplementary Figure 7: ﻿Clonal sharing between clusters informs differentiation.**

**A,** Proportions of cell states in pre-existing clones, defined as expanded clonotypes shared in screening and M2D1. **B,**Proportions of cell states in pre-existing clones, defined as expanded clonotypes shared between M2D1 and M7D1. **C,** Cell cycle in distinct CD8+ T cell subsets in expanded clonotypes shared between screening and M7D1. Scoring for S phase. p = 0.57, p = 0.63, p = 0.28 and p = 0.31 by Wilcoxon test, respectively. **D,** RNA velocity projected on UMAP. Colors denote cell states. **E,**Root cells and endpoints of RNA velocity analysis. **F,** Velocity as in **D**. Cells of clonotype 2 are highlighted. **G,** Proportion of cells with singleton clonotypes in CAR-T cells and endogenous CD8+ T cells at screening, M2D1 and M7D1 from full-length scRNA-Seq sequencing approach. **H,** Singleton percentage for long-term responder (LTR) versus short-term responder (STR) patients at different timepoints based on BM and PB samples of 24 patients with full-length scRNA-Seq approach. *p = 0.037 at M2D1 by Wilcoxon test. **I,** Shannon diversity index across timepoints based on BM and PB samples of 24 patients with full-length scRNA-Seq approach. *p = 0.012 for CART vs. Screening, **p = 0.0096 for M2D1 vs. M7D1 by Wilcoxon test. **J,** Shannon diversity index for LTR versus STR patients at different timepoints based on BM and PB samples of 24 patients with full-length scRNA-Seq approach. ****p < 2.2e-16 for screening, M2D1 and M7D1, ****p < 4.7e-9 for CART by Wilcoxon test.

**
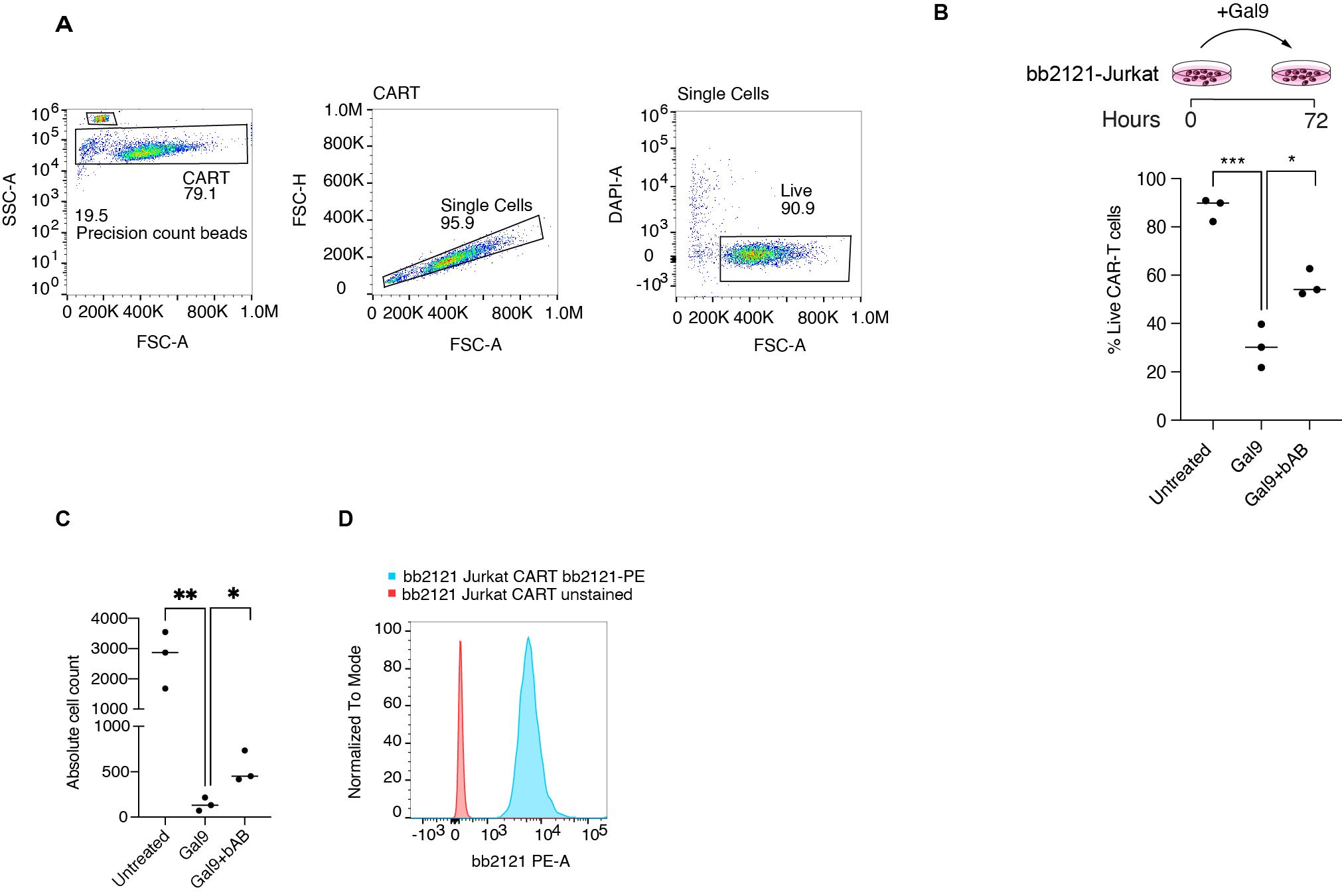
 Supplementary Figure 8: GAL9 treatment results in reduced viability in CAR-T cells.**

**A,** Gating strategy for flow cytometric measurement of cell viability. **B,** Percentage of live CAR-T cells following treatment with Gal9 and blocking antibody (bAb). ***p = 0.0006 and *p = 0.0132 by unpaired t-test, respectively. **C,** Absolute CAR-T cell count following treatment with Gal9 and blocking antibody (bAb). **p = 0.0094 and *p = 0.0221 by unpaired t-test, respectively. **D,** bb2121 staining on CAR-T cells.

**References**

1. An Efficacy and Safety Study of bb2121 in Subjects With Relapsed and Refractory Multiple Myeloma and in Subjects With High-Risk Multiple Myeloma (KarMMa-2). <https://clinicaltrialsgov/ct2/show/NCT03601078>.

2. Munshi NC, Anderson LD, Jr., Shah N, Madduri D, Berdeja J, Lonial S, et al. Idecabtagene Vicleucel in Relapsed and Refractory Multiple Myeloma. N Engl J Med. 2021;384(8):705-16.

3. Efficacy and Safety Study of bb2121 Versus Standard Regimens in Subjects With Relapsed and Refractory Multiple Myeloma (RRMM) (KarMMa-3). 1 <https://clinicaltrialsgov/ct2/show/NCT03651128?cond=NCT03651128&draw=2&rank=1>.

4. Rodriguez-Otero P, Ailawadhi S, Arnulf B, Patel K, Cavo M, Nooka AK, et al. Ide-cel or Standard Regimens in Relapsed and Refractory Multiple Myeloma. N Engl J Med. 2023;388(11):1002-14.

5. Picelli S, Faridani OR, Björklund AK, Winberg G, Sagasser S, Sandberg R. Full-length RNA-seq from single cells using Smart-seq2. Nature protocols. 2014;9(1):171-81.

6. Stoeckius M, Zheng S, Houck-Loomis B, Hao S, Yeung BZ, Mauck WM, 3rd, et al. Cell Hashing with barcoded antibodies enables multiplexing and doublet detection for single cell genomics. Genome Biol. 2018;19(1):224.

7. Stoeckius M, Hafemeister C, Stephenson W, Houck-Loomis B, Chattopadhyay PK, Swerdlow H, et al. Simultaneous epitope and transcriptome measurement in single cells. Nat Methods. 2017;14(9):865-8.

8. Im NG, Guillaumet-Adkins A, Wal M, Rogers AJ, Frede J, Havig CC, et al. Regulatory Programs of B-cell Activation and Germinal Center Reaction Allow B-ALL Escape from CD19 CAR T-cell Therapy. Cancer Immunol Res. 2022;10(9):1055-68.

9. Anand P, Guillaumet-Adkins A, Dimitrova V, Yun H, Drier Y, Sotudeh N, et al. Single-cell RNA-seq reveals developmental plasticity with coexisting oncogenic states and immune evasion programs in ETP-ALL. Blood. 2021;137(18):2463-80.

10. Bolger AM, Lohse M, Usadel B. Trimmomatic: a flexible trimmer for Illumina sequence data. Bioinformatics. 2014;30(15):2114-20.

11. Dobin A, Davis CA, Schlesinger F, Drenkow J, Zaleski C, Jha S, et al. STAR: ultrafast universal RNA-seq aligner. Bioinformatics. 2013;29(1):15-21.

12. Li B, Dewey CN. RSEM: accurate transcript quantification from RNA-Seq data with or without a reference genome. BMC Bioinformatics. 2011;12:323.

13. Anders S, Pyl PT, Huber W. HTSeq--a Python framework to work with high-throughput sequencing data. Bioinformatics. 2015;31(2):166-9.

14. Aran D, Looney AP, Liu L, Wu E, Fong V, Hsu A, et al. Reference-based analysis of lung single-cell sequencing reveals a transitional profibrotic macrophage. Nat Immunol. 2019;20(2):163-72.

15. Fernández JM, de la Torre V, Richardson D, Royo R, Puiggròs M, Moncunill V, et al. The BLUEPRINT Data Analysis Portal. Cell Syst. 2016;3(5):491-5.e5.

16. Bunis DG, Andrews J, Fragiadakis GK, Burt TD, Sirota M. dittoSeq: Universal User-Friendly Single-Cell and Bulk RNA Sequencing Visualization Toolkit. Bioinformatics. 2020;36(22-23):5535-6.

17. Korotkevich G SV, Sergushichev A (2019). “Fast gene set enrichment analysis.” bioRxiv. doi:10.1101/060012, <http://biorxiv.org/content/early/2016/06/20/060012>.

18. Anderson ND, Birch J, Accogli T, Criado I, Khabirova E, Parks C, et al. Transcriptional signatures associated with persisting CD19 CAR-T cells in children with leukemia. Nat Med. 2023;29(7):1700-9.

19. Efremova M, Vento-Tormo M, Teichmann SA, Vento-Tormo R. CellPhoneDB: inferring cell-cell communication from combined expression of multi-subunit ligand-receptor complexes. Nat Protoc. 2020;15(4):1484-506.

20. Young MD, Behjati S. SoupX removes ambient RNA contamination from droplet-based single-cell RNA sequencing data. Gigascience. 2020;9(12).

21. Hao Y, Hao S, Andersen-Nissen E, Mauck WM, 3rd, Zheng S, Butler A, et al. Integrated analysis of multimodal single-cell data. Cell. 2021;184(13):3573-87 e29.

22. Song L, Cohen D, Ouyang Z, Cao Y, Hu X, Liu XS. TRUST4: immune repertoire reconstruction from bulk and single-cell RNA-seq data. Nat Methods. 2021;18(6):627-30.

23. Borcherding N, Bormann NL, Kraus G. scRepertoire: An R-based toolkit for single-cell immune receptor analysis. F1000Res. 2020;9:47.

24. La Manno G, Soldatov R, Zeisel A, Braun E, Hochgerner H, Petukhov V, et al. RNA velocity of single cells. Nature. 2018;560(7719):494-8.
